# Gene Expression Profiles Identify Biomarkers of Resistance to Decitabine in Myelodysplastic Syndromes

**DOI:** 10.3390/cells10123494

**Published:** 2021-12-10

**Authors:** Seungyoun Kim, Dong-Yeop Shin, Dayeon Kim, Somi Oh, Junshik Hong, Inho Kim, Eunju Kim

**Affiliations:** 1 Division of Radiation Biomedical Research, Korea Institute of Radiological and Medical Sciences, Seoul 01812, Korea; sksgml4488@gmail.com (S.K.); kimd1106@daum.net (D.K.); 2 Department of Radiological and Medico-Oncological Sciences, University of Science and Technology, Daejeon 34113, Korea; 3 Cancer Research Institute, Seoul National University College of Medicine, Seoul 03080, Korea; shindongyeop@snuh.org (D.-Y.S.); sororom@hanmail.net (S.O.); alertjun@hanmail.net (J.H.); 4 Center for Medical Innovation, Biomedical Research Institute, Seoul National University Hospital, Seoul 03080, Korea; 5 Division of Hematology and Medical Oncology, Department of Internal Medicine, Seoul National University Hospital, Seoul 03080, Korea

**Keywords:** myelodysplastic syndromes, decitabine, resistance, biomarkers

## Abstract

Myelodysplastic syndrome (MDS) is a clonal hematopoietic stem cell disease characterized by inefficient hematopoiesis and the potential development of acute leukemia. Among the most notable advances in the treatment of MDS is the hypomethylating agent, decitabine (5-aza-2′deoxycytidine). Although decitabine is well known as an effective method for treating MDS patients, only a subset of patients respond and a tolerance often develops, leading to treatment failure. Moreover, decitabine treatment is costly and causes unnecessary toxicity. Therefore, clarifying the mechanism of decitabine resistance is important for improving its therapeutic efficacy. To this end, we established a decitabine-resistant F-36P cell line from the parental F-36P leukemia cell line, and applied a genetic approach employing next-generation sequencing, various experimental techniques, and bioinformatics tools to determine differences in gene expression and relationships among genes. Thirty-eight candidate genes encoding proteins involved in decitabine-resistant-related pathways, including immune checkpoints, the regulation of myeloid cell differentiation, and PI3K-Akt signaling, were identified. Interestingly, two of the candidate genes, AKT3 and FOS, were overexpressed in MDS patients with poor prognoses. On the basis of these results, we are pursuing development of a gene chip for diagnosing decitabine resistance in MDS patients, with the goal of ultimately improving the power to predict treatment strategies and the prognosis of MDS patients.

## 1. Introduction

Myelodysplastic syndrome (MDS) is a hematologic disorder characterized by marrow failure and a risk of clonal progression. MDS, also known as pre-leukemia, is characterized by a high risk of transformation to acute myeloid leukemia (AML). The exact cause of MDS is uncertain in many cases, although preceding exposure to radiation therapy, chemotherapy, poisonous solvents, tobacco, or agricultural chemicals are risk factors in some cases [1]. Apart from allogeneic hematopoietic stem cell transplantation (HSCT), effective therapies are absent; thus, MDS has long been considered a difficult to control disease. Although allogeneic HSCT can be a curative treatment option for MDS, high treatment-related mortality limits the universal application of allogeneic HSCT in all patients with MDS. This has led to the development of the International Prognostic Scoring System (IPSS), a risk stratification strategy for predicting prognosis and selecting appropriate patients who might benefit from allogeneic HSCT.

The IPSS classifies patients into four risk categories: low, intermediate-1, intermediate-2, and high. In line with EMEA (Europe, the Middle East, and Africa) policy, IPSS is still used to obtain drug approval in some European countries and is widely used in clinics [2], but it often lacks the ability to accurately discriminate between lower-risk patients (low vs. intermediate-1) and higher-risk patients (intermediate-2 vs. high). For this reason, a revised classification has been introduced that includes a refined cytogenetic classification and definition of cytopenia, and an improved classification according to bone marrow blastosis [3]. The revised IPSS (IPSS-R) stratifies patients into five subgroups: very low, low, intermediate, high, and very high risk. Treatment intent for patients with MDS differs according to the risk stratification. In lower-risk patients, treatment is usually focused on improving the quality of life of patients by decreasing transfusion needs and preventing leukemic transformation. In higher-risk patients, the treatment goal is to prolong survival by preventing transformation into AML [4].

The most notable progress in the treatment of MDS is the development of the hypomethylating agents (HMAs) 5-azacytidine (AZA) and 5-aza-2′deoxycytidine (decitabine), which were licensed by the US Food and Drug Administration (FDA) in 2004 and 2006, respectively. The mode of action of HMAs is to inhibit DNA methyltransferases (DNMTs), especially at lower doses [5,6,7]. Objective outcomes, including complete responses (CRs) and partial responses (PRs), are reported in approximately 15% to 20% of patients, and an additional 20% to 30% of patients achieve hematologic improvement [8,9,10]. Moreover, a minority of patients on long-term HMA treatment can achieve progression-free survival for several years. However, most patients experience disease progression owing to the development of resistance to HMA agents during treatment [11]. In such cases, there is no standard therapeutic alternative [12]. Thus, elucidating the mechanisms underlying resistance to HMAs, which are currently incompletely understood [13], is essential for the development of effective targeted therapies.

Decitabine (DEC) is known to exert an inhibitory effect on DNA methylation as well as block DNA replication and the induction of apoptosis [14,15]. Several studies confirmed that DEC targets 14,000 regulatory regions of genes in different cancer cell types as a DNA methyltransferase inhibitor (DNMTi) [16]. DEC demonstrates broad effects across most patient subgroups in various risk categories. A recent study also revealed that DEC shows efficacy in patients with TP53-mutated MDS [17]. The labeled usage of DEC is more convenient than that of AZA because the duration of treatment per cycle is shorter for DEC (5 days) than AZA (7 days). Despite these benefits of DEC over AZA, no second-line therapy has been established for patients who are resistant to DEC. The prognosis is still poor after the failure of DEC treatment, with a median survival of about 4–5 months [18,19]. Recent findings demonstrated that molecular data represent a biological opportunity for improving patient response rates and outcomes, as clinical variables and patient characteristics do not consistently predict response to DEC [20,21,22].

The aim of this study is to identify genetic profiles that can be translated to potential approaches or biomarkers for the diagnosis and therapy of MDS patient with resistance to DEC. In this study, we established a DEC-resistant cell line (F-36P/DEC) from the F-36P human leukemia (MDS) cell line, and investigated the mechanism of resistance to DEC using gene expression profiling. In addition, we confirmed major deregulated genes and pathways through our experimental and analytic system. Our findings shed light on the mechanism of resistance to DEC and provide insights into the development of novel strategies for the prognostic prediction and personalized therapy of MDS patients.

## 2. Materials and Methods

### 2.1. DEC-Resistant Cell Selection and Culture

The DEC-resistant F-36P cell line, F-36P/DEC, was established from the parental F-36P cell line [23] (European Collection of Authenticated Cell Cultures (ECACC), Salisbury, UK) by continuously exposing parental F-36P cells to gradually increasing concentrations of DEC (Sigma-Aldrich, St. Louis, MO, USA). The initial DEC concentration was 1 μmol/L and was then increased exponentially in steps until reaching 32 μmol/L. Selected cells were cultured in DEC-free medium for at least 2 weeks prior to experiments. F-36P and F-36P/DEC cells were incubated in RPMI-1640 medium (GenDEPOT, Katy, TX, USA) containing 5% fetal bovine serum (GenDEPOT), 1% penicillin (GenDEPOT), 5 ng/mL interleukin (IL)-3 (Sigma-Aldrich), and 2 mM glutamine (Sigma-Aldrich) at 37 °C in a humidified, 5% CO_2_ atmosphere.

### 2.2. Cell Morphology and Measurement of Drug Sensitivity

F-36P and F-36P/DEC cells were observed under an inverted light microscope (Nikon, Melville, NY, USA) equipped with a 10× eyepiece and both 10× and 40× objective lenses. Images of each cell type were acquired using Motic Images Plus 2.0 software (Motic Inc., Xiamen, China). Cells were seeded into 96-well plates at 1 × 10^4^ cells/well in 0.1 mL of complete medium. The proliferation and cytotoxicity of DEC were investigated using WST assay kits (LPS Solution, Daejeon, Korea). After incubation in 96-well plates, F-36P and F-36P/DEC cells were incubated without or with different concentrations of DEC (1.75, 3.5, 7.5, 15, and 30 μmol/L) for 72 h. Cell proliferation was measured by adding 20 μL of WST reagent to each well and then incubating for 1 h in a 37 °C incubator. The absorbance of wells at 450 nm was read using a Multiskan FC instrument (Thermo Fisher Scientific Inc., Waltham, MA, USA), and the percentage growth was calculated. Proliferation was assessed at 24, 48, and 72 h in wells without DEC. The method for measuring cell cytotoxicity at 72 h following the seeding of cells was identical to that used to measure cell proliferation. The concentration of DEC required to inhibit growth by 50% (half-maximal inhibitory concentration) at 72 h was used for calculation of IC_50_. The degree of resistance was scored relative to the IC_50_ value. The IC_50_ value of DEC was analyzed by probit analysis using SPSS software version 26.0 (IBM Inc., Armonk, NY, USA).

### 2.3. Fluorescence-Activated Cell Sorting (FACS) Analysis

A cell-cycle analysis was conducted using a BD Accuri C6 Plus flow cytometer (BD Biosciences, Franklin Lakes, NJ, USA). Briefly, cells were seeded into six-well plates at 2 × 10^4^ cells/well in 2 mL. After 72 h, cells were harvested by centrifugation at 1000× *g* for 5 min at room temperature, washed with PBS, fixed overnight at 4 °C with ice-cold 75% ethanol, and incubated with 10 μg/mL RNase A (Sigma-Aldrich) and 20 μg/mL propidium iodide (Sigma-Aldrich) for 15 min at room temperature in the dark. Cells in G1, S, and G2/M phases were identified based on fluorescence intensity, and the cell-cycle distribution was analyzed using BD Accuri C6 Plus software version 1.0.23.1 (BD Biosciences).

### 2.4. RNA Isolation

Total RNA was isolated using QIAzol reagent (Qiagen, Hilden, Germany). RNA quality was assessed with an Agilent 2100 bioanalyzer using an RNA 6000 Nano Chip (Agilent Technologies, Amstelveen, Netherlands), and RNA quantity was determined using a NanoDrop 2000 Spectrophotometer (ND-2000; Thermo Fisher Scientific Inc., Waltham, MA, USA).

### 2.5. NanoString Targeted Gene Expression

Total RNA samples were analyzed using the NanoString nCounter PanCancer Pathways Panel Analysis System (NanoString Technologies, Seattle, WA, USA). For each hybridization reaction, 100 ng total RNA (or any quantity that was present in a 5 µL aliquot of purified RNA if less than 100 ng) was used. nCounter raw data (RCC files) for each sample were imported into nSolver Analysis Software version 4.0 (NanoString Technologies) for review of quality control metrics (all passed). The spike-in positive control geometric mean normalization factor (without negative control background subtraction) was determined for each sample using nSolver. Raw counts from these genes, together with spike-in positive controls, were then used to perform both positive control and reference gene normalization of the raw data in nSolver. These data were then exported in CSV format for gene expression analyses. Count data were converted into counts per million mapped reads (CPM) using the fpm function in DESeq2. A pseudo count of 1 was added to all counts before calculating the log_2_ value and the log_2_ fold-change was calculated. Data mining and graphic visualization were performed using nCounter Advanced Analysis software (NanoString Technologies).

### 2.6. Gene and Pathway Enrichment Analyses of Differentially Expressed Genes (DEGs)

DAVID (Database for Annotation, Visualization and Integrated Discovery; https://david.ncifcrf.gov/, accessed on 13 November 2020), an online biological information database, was used for the pathway enrichment analysis of DEGs. The Gene Ontology (GO) term enrichment analysis annotated by the DAVID database is composed of three attributes: molecular function (MF), biological process (BP), and cellular component (CC). Pathway enrichment analyses were conducted using the DAVID website tools, Kyoto Encyclopedia of Genes and Genomes (KEGG), and REACTOME; *p*-values < 0.05 were considered statistically significant.

### 2.7. Protein–Protein Network and Module Analysis

Protein–protein interaction (PPI) networks were mapped using Cytoscape (version 3.8.2; https://cytoscape.org/, accessed on 11 November 2020), a public-access software that can graphically edit, display, and analyze the network. Significant modules in PPI networks were identified using Molecular Complex Detection (MCODE), a plug-in app of Cytoscape designed to analyze densely connected regions by clustering a given network. Hub genes were identified using the cytoHubba analysis in Cytoscape. Analyses with ClueGO, a Cytoscape plug-in, were performed using databases updated in May 2021.

### 2.8. Quantitative Reverse Transcription-Polymerase Chain Reaction (qRT-PCR)

Total RNA was extracted using QIAzol reagent (Qiagen), then reverse transcribed into cDNA using amfiRivert reverse transcriptase (GenDEPOT) according to the manufacturer’s instructions. cDNA was amplified by PCR on a Mic Real-Time PCR system (Bio Molecular Systems, Upper Coomera, QLD, Australia) using Luna Universal qPCR master mix (New England Biolabs Inc., Ipswich, MA, USA) and primer pairs specific to target genes (Table S1). Expression data were normalized to the geometric mean of the housekeeping gene, GAPDH, to control the variability in expression levels and analyzed using the ΔCT and 2^−ΔΔCT^ quantification method.

### 2.9. Validation of Genetic Alterations in Candidate Genes

Genetic alterations in candidate genes in the myeloid neoplasm dataset were analyzed using cBioPortal (http://cbioportal.org/, accessed on 1 October 2021), an online analysis platform for multidimensional cancer genomic data that provides a collective visualization of genes, samples, and data types.

### 2.10. Patient Enrollment and Treatment–Bone Marrow Samples

Bone marrow-derived blood samples were obtained from four MDS patients diagnosed at Seoul National University Hospital (SNUH). Patient samples were collected with each patient’s informed consent after receiving approval from the Institutional Review Board of SNUH. MDS subtypes were classified according to the revised World Health Organization classification of myeloid neoplasm [24]. Patients were treated with DEC (20 mg/m^2^/d for 5 days every 4 weeks), and response to treatment and clinical outcome were evaluated according to revised International Working Group (IWG) response criteria. The patients used for the analysis were classified into two groups: (1) Patients 1 and 2, bone marrow samples obtained at initial diagnosis, pre-DEC treatment; and (2) Patients 3 and 4, samples obtained in complete response (CR) status after four cycles of DEC treatment.

### 2.11. Statistical Analysis

All experiments were performed in at least triplicate (*n* ≥ 3), and data are presented as means ± standard deviation (SD). Differences between two sample means were determined with a Student’s *t*-test for independent samples using SPSS software version 26.0. Differences with *p*-values < 0.05 were considered statistically significant.

## 3. Results

### 3.1. Establishment of the DEC-Resistant Cell Line, F-36P/DEC

**Figure 1:**
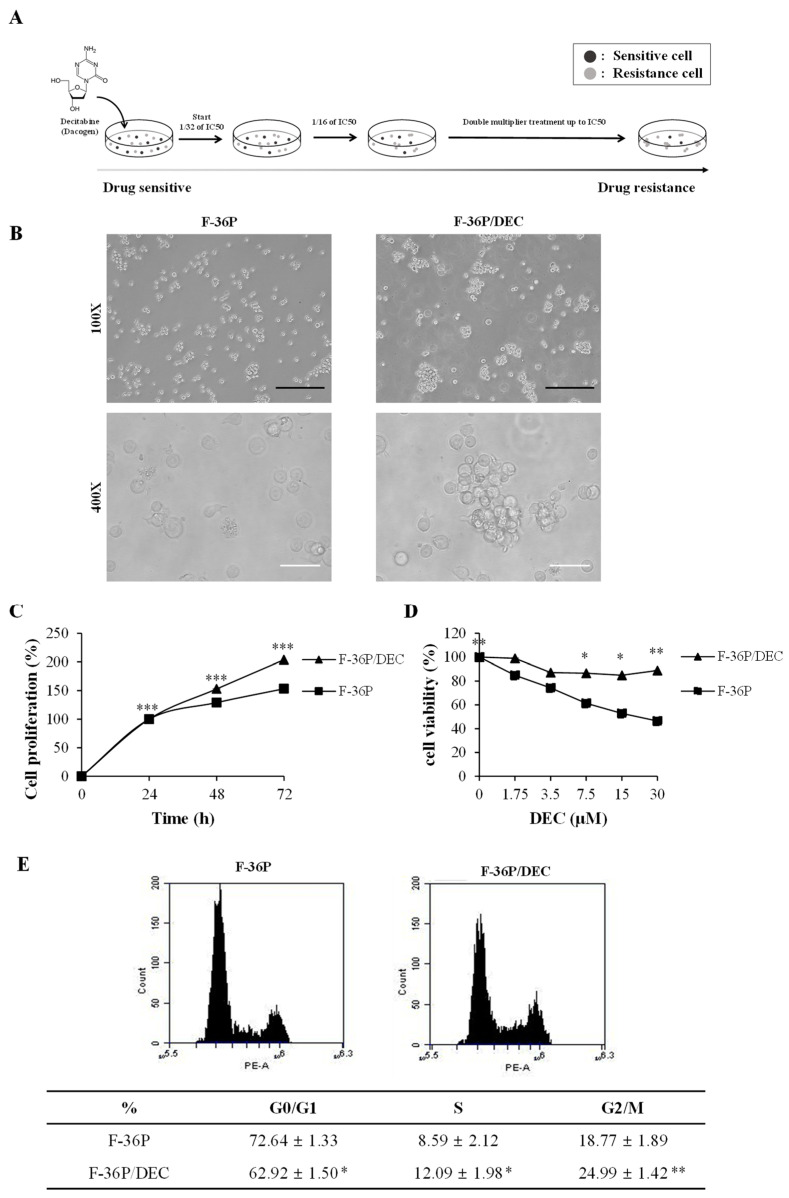
Validation of DEC resistance in the F-36P/DEC cell line. (**A**) Schematic showing how parental F-36P cells were used to create cells DEC-resistant F-36P/DEC cells. (**B**) Morphological observations of F-36P and F-36P/DEC cells. Magnification, 100× and 400×. Black scale bar, 250 μm; white scale bar, 50 μm. (**C**) The proliferation of F-36P and F-36P/DEC cells was analyzed by cell counting using WST assay kits and compared. (**D**) The viability of DEC-treated F-36P and F-36P/DEC cells was measured at 72 h using a WST assay and compared. (**E**) The cell-cycle distribution of F-36P and F-36P/DEC cells was measured by flow cytometry using propidium iodide (PI) and the G2/M phase ratio was determined and compared. Results are presented as means ± SD (error bars) (*n* = 3; * *p* < 0.05, ** *p* < 0.01, *** *p* < 0.001 compared to the control).

To study the mechanism of resistance to DEC, we established an in vitro DEC-resistant cell line model from the parental cell line F-36P (Figure 1A). The morphological differences between F-36P cells and the derived DEC-resistant F-36P/DEC cells were surveyed using an inverted light microscope at magnifications of 100× and 400×. As shown in Figure 1B, F-36P cells were detected as single cells under the light microscope, whereas F-36P/DEC cells were observed to aggregate. We further found that F-36P/DEC cells stably proliferated over 72 h (Figure 1C). The IC_50_ value for DEC was 17.27 μmol/L in F-36P cells and more than 30 μmol/L in F-36P/DEC cells. However, although we used DEC at concentrations up to 1000 μmol/L in the F-36P/DEC cell line, we were unable to accurately calculate an IC_50_ value for these cells (Figure 1D). To further explore the biological properties of F-36P/DEC cells, we compared the cell-cycle properties of F-36P/DEC cells with those of F-36P cells, finding that the proportion of cells in the G2/M phase of the cell cycle was significantly increased in F-36P/DEC cells (Figure 1E). Collectively, these results indicate that the F-36P/DEC cell model generated for these studies is significantly resistant to DEC and has characteristics that differ from those of F-36P cells.

### 3.2. Identification of Genes That Are Differentially Expressed between F-36P/DEC and F-36P Cells

**Figure 2:**
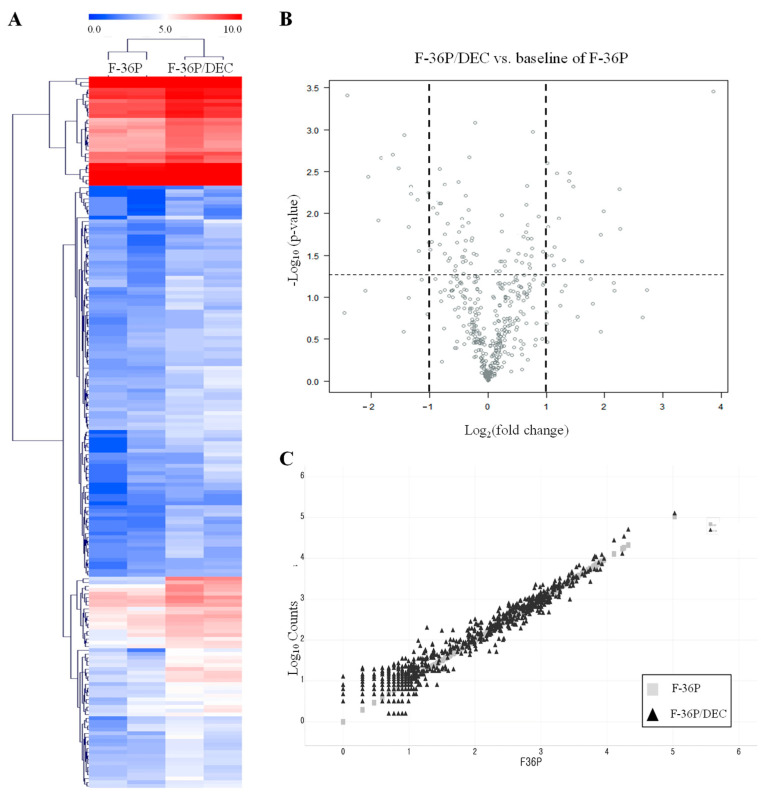
Analysis of DEGs between F-36P/DEC and F-36P cells. (**A**) Heatmap illustrates significant DEGs between F-36P cells and F-36P/DEC cells. Representations of genes were processed using the general linear model likelihood ratio test (*p* < 0.05 and absolute log_2_ fold change > 1). (**B**) Significant DEGs between F-36P cells and F-36P/DEC cells, in the form of log_10_ (*p*-value) versus log_2_ (fold change), are presented graphically as volcano plots. The horizontal lines indicate statistical significance at *p* < 0.05. (**C**) Scatter plot of DEGs in F-36P/DEC cells compared with those in F-36P cells. DEGs were selected on the basis of (logFC) ≥ 1.5 and *p* < 0.05.

To identify genes associated with DEC resistance, we screened for candidate genes that were differentially expressed between F-36P and F-36P/DEC cells using a NanoString analysis [25]. The 189 differentially expressed genes (DEGs) obtained as a result of this analysis were expressed as a cluster heatmap [26] using nSolver (Figure 2A and Table S2). For a cluster heatmap, hierarchical clustering using average linkage and a Euclidean distance metric was used. A volcano plot used to display the distribution of DEGs in F-36P/DEC relative to F-36P (Figure 2B and Table S3) shows −log_10_ (*p*-value) values and log_2_ fold changes in DEGs. Statistically significant genes (*p*-value ≤ 0.05) are located at the top of the plot above the horizontal lines, with strongly differentially expressed genes falling on either side. Using a scatter plot to show the distribution of F-36P/DEC DEGs, we found that the larger the difference in expression level, the more the trend line deviated from a linear function (Figure 2C). Collectively, these data allowed us to select more specific DEGs between F-36P/DEC and F-36P cells. We also performed a gene ontology (GO) analysis [27] of significantly enriched genes to obtain overview information about the function of protein products of our DEGs (Table 1). The regulation of transcription, cell cycle, and proliferation pathways (biological process), nucleus and extracellular region (cellular compartment), and transcription factor binding and protein kinase activity (molecular function) were the GO properties most frequently associated with identified DEGs.

### 3.3. Functional Classification of DEGs Associated with DEC Resistance of the Cell Line, F-36P/DEC

**Figure 3:**
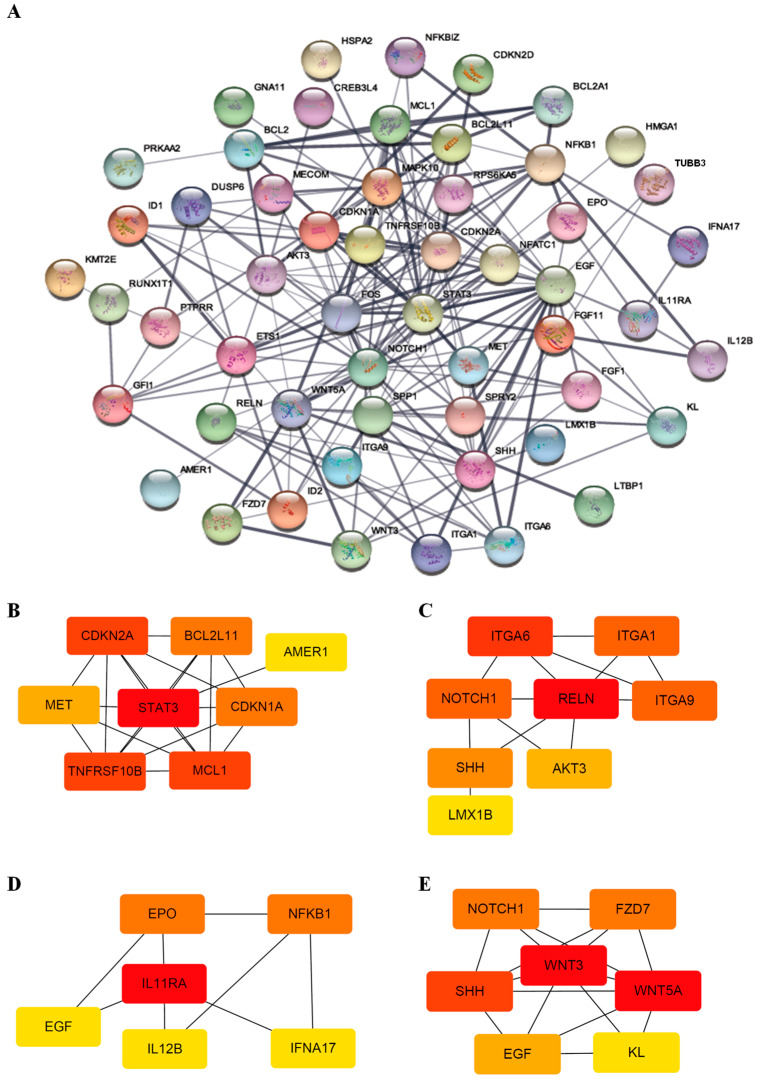
PPI network and subnetworks generated from DEGs. (**A**) The PPI network was processed using the STRING plug-in of the Cytoscape program (version 3.8.2). Each circle represents a gene (node), and connections between circles (edges) represent direct or indirect interactions. Of the 66 genes that were differentially expressed between F-36P cells and F-36P/DEC cells, 54 were functionally linked with 198 edges. PPI enrichment *p*-values < 0.04 were considered significant. (**B–E**) Module analyses. Module clusters were extracted using MCODE and cytoHubba analyses. Hub genes are indicated in red, and co-expressed genes are indicated in orange, yellow, or blue according to their degree of importance. Module 1 (**B**) contains 8 nodes and 20 edges. Module 2 (**C**) contains 8 nodes and 13 edges. Module 3 (**D**) contains 6 nodes and 8 edges. Module 4 (**E**) contains 7 nodes and 16 edges.

**Figure 4:**
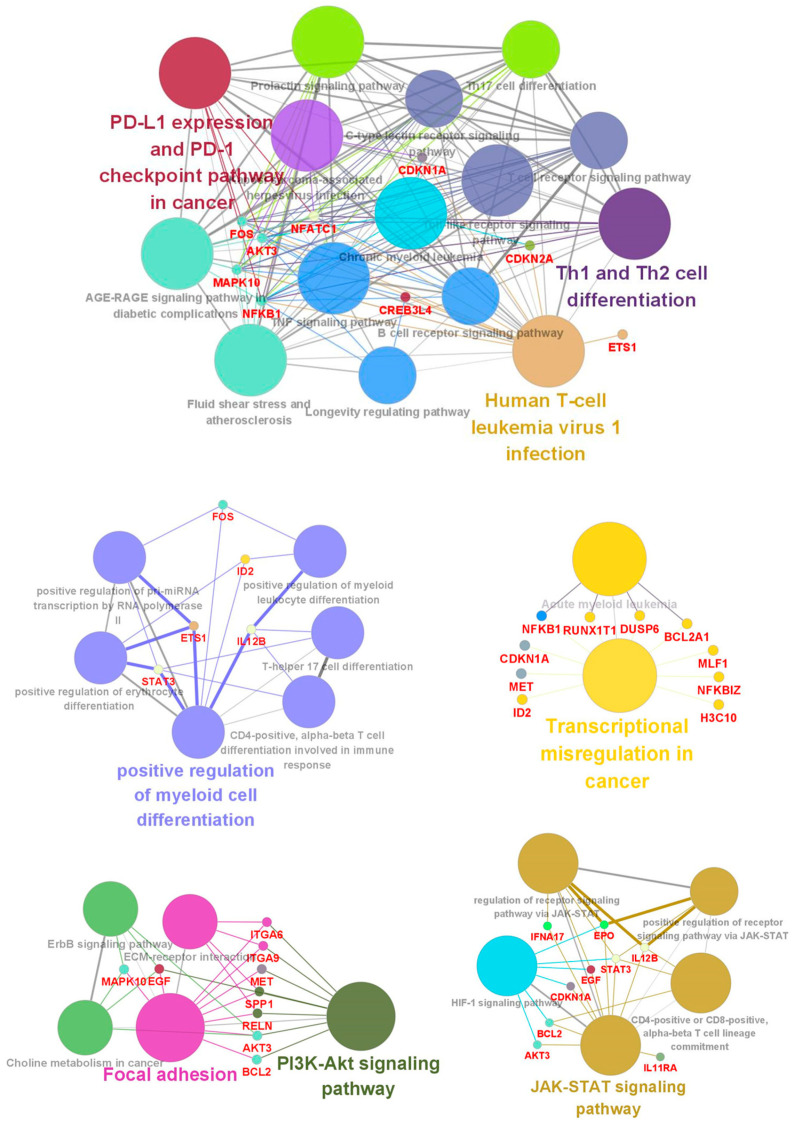
ClueGo analysis-based enrichment maps derived from GO terms associated with DEGs. Highly interconnected GO terms are presented. Terms in bold font indicate top GO terms. Gene names within subgroups were generated using ClueGO default settings. All GO terms shown are statistically significant (*p* < 0.05 with Bonferroni correction).

Protein–protein interaction (PPI) networks can reveal physical contacts between protein pairs and identify biological pathway clusters [28]. Using this approach, we generated a PPI network from 66 DEGs that contained 54 nodes and 198 edges (Figure 3A and Table S4). The resulting network was still very complex; thus, we subsequently performed a minimal common oncology data elements (MCODE) analysis with specific modules [29]. The networks were also confirmed by cytoHubba analysis [30], which generated similar hub networks (Figure 3B–E). This subnetwork analysis identified *STAT3* (signal transducer and activator of transcription 3), *RELN* (reelin), *IL11RA* (interleukin-11 receptor, alpha subunit), and *WNT3* (proto-oncogene protein Wnt-3), among others, as hub genes. A ClueGO and module analysis, which can improve biological interpretation by organizing separate GO pathway term networks [31,32], revealed various additional processes linked to DEC resistance, including immune cell activation, regulation of myeloid cell differentiation, transcriptional misregulation, PI3K-Akt signaling, and JAK-STAT signaling (Figure 4).

### 3.4. Validation of Candidates Identified by RNA-Seq Analysis

**Figure 5:**
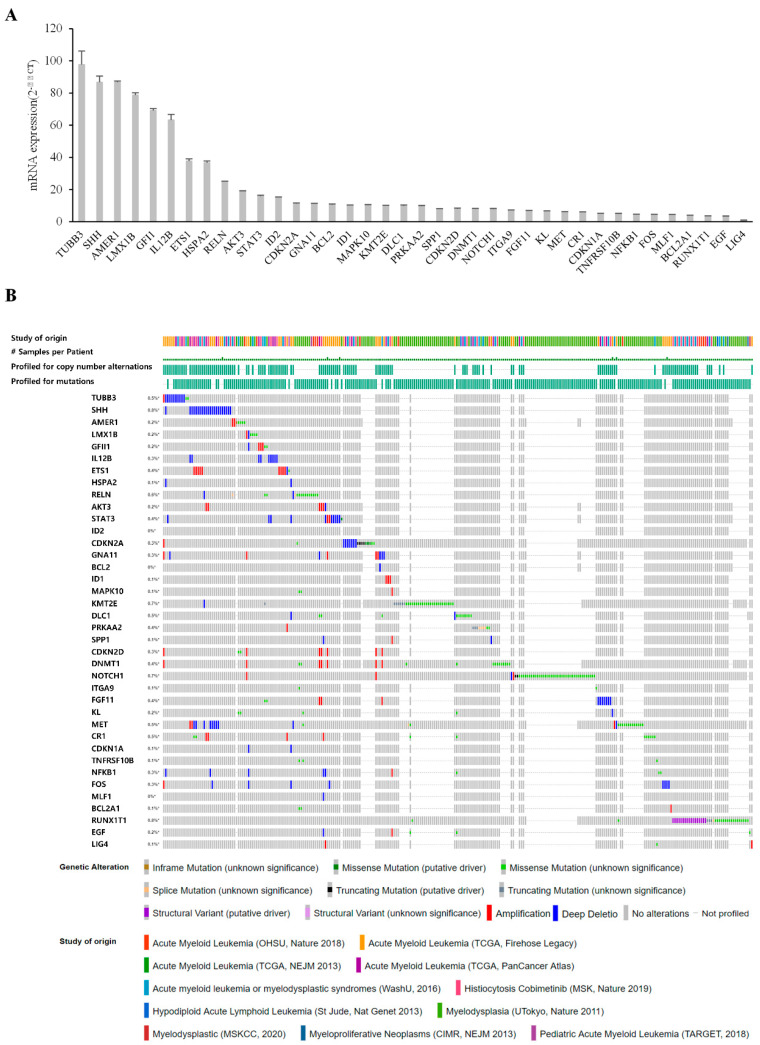
Verification of candidate genes by qRT-PCR and cBioPortal analysis. (**A**) The graph depicts mRNA levels of candidate genes that were differentially expressed between F-36P cells and F-36P/DEC cells. All candidate genes were significantly upregulated in F-36P/DEC cells. Differential expression is considered significant at *p* < 0.05. Error bars indicate standard deviations (*n* = 3). (**B**) Myeloid neoplasm datasets (http://cbioportal.org/, accessed on 1 October 2021) were interrogated for genetic alterations in candidate genes. Alterations were found in 0% to 0.8% of the respective analyses and are depicted graphically. Many candidate genes exhibited amplification alterations.

We next verified gene expression profiles using quantitative RT-PCR (qRT-PCR) analysis, selecting the top 38 genes with the highest fold-changes for validation. These top genes included those encoding proteins involved in immune cell activation (GFI1, IL12B, NFKB1, FOS), the regulation of myeloid cell differentiation (ETS1, ID2, KMT2E), transcriptional misregulation (BCL2A1, MLF1, RUNX1T1), PI3K-Akt signaling (AKT3, MET, RELN), and the JAK-STAT signaling pathway (BCL2, EGF, STAT3). The molecular properties of these gene products are summarized in the Supplementary Data (Table S5). As shown in Figure 5A, qRT-PCR analyses confirmed upregulation of our candidates in F-36P/DEC cells. Using the cBioPortal for cancer genomics, originally developed for interactive exploration of multidimensional cancer genomic datasets [33], we extracted a dataset from the clinical expression dataset of myeloid neoplasms containing 11 studies and 6940 patient samples (Figure 5B). The majority of our candidates were found in the extracted dataset, consolidating the credibility of our candidate list. Thirty-eight genes have been shown to be associated with different types of alterations, most of which have been associated with mutations. In particular, 24 genes were associated with amplification (red bars). Therefore, these results suggest that most of our candidate genes are clinically relevant and allow reliable discovery.

### 3.5. Comparison of Gene Expression in Bone Marrow from Patients with MDS

**Figure 6:**
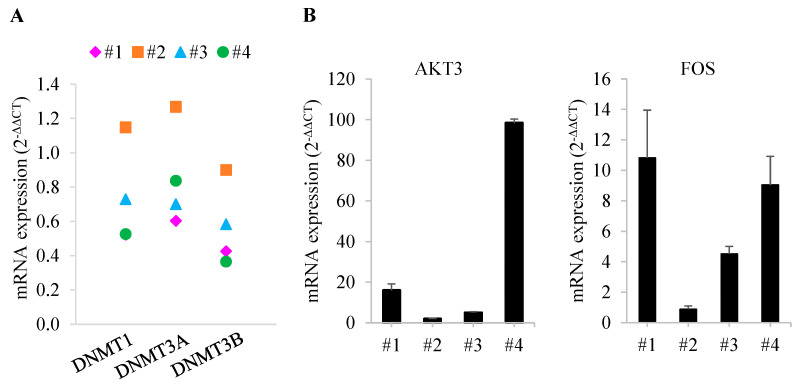
Comparison of gene expression in MDS patients with and without DEC treatment. (**A**) Comparison of DNMT1, DNMT3A, and DNMT3B mRNA expression levels by patient. (**B**) Confirmation of differences in mRNA expression of AKT3 and FOS for each patient. All differences in expression are significant at the *p* < 0.05 level. Error bars indicate SD (*n* = 3).

Four patients with MDS were enrolled in this study. The median age of patients at the time of diagnosis of MDS was 64 years (range, 46–74). MDS subtypes were MDS-EB1 and MDS-EB2, and IPSS risk categories at baseline were intermediate-2 and high risk (Table 2). Patients were treated with a median of nine cycles of DEC (range, 2–16), and all patients achieved an objective response (complete remission in three patients and partial remission in one patient). The duration of the response was less than 1 year in two patients (9 months in Patient 1 and 11 months in Patient 3); another patient (Patient 4) proceeded to HSCT after five cycles of DEC, but expired due to pneumonia 17 months later. In contrast to the relatively shorter survival of these patients, Patient 2 showed a significantly longer survival (52 months). IPSS-R was well correlated with the time to progression, with two patients with very high risk by IPSS-R progressing within 1 year, and two patients with intermediate and high risk by IPSS-R showing continued response to DEC after 2 years and more than 4 years, respectively. However, IPSS and the MDS subtypes MDS-EB1 and MDS-EB2 were not predictive of prognosis. We next performed qRT-PCR to assess gene expression in patient bone marrow samples, using bone marrow-derived samples before DEC treatment for Patients 1 and 2 and samples obtained after four cycles of DEC treatment for Patients 3 and 4. For this analysis, qRT-PCR results for each bone marrow sample, quantified using the 2^−ΔΔCT^ method, were normalized to those of 10 normal bone marrow samples (defined as 1). This analysis confirmed differences in mRNA expression of DNA methyltransferase (DNMT) genes in patient bone marrow samples, specifically showing a slightly higher expression of DNMT1 and DNMT3A in Patient 2 compared with normal bone marrow, but no difference in DNMT3B. In other patients, the expression of DNMT genes was decreased compared to that of normal controls (Figure 6A). Surprisingly, a comparison of the mRNA levels of 38 genes in bone marrow samples from patients showed that expression levels of AKT3 and FOS genes were similar to normal in Patient 2 but increased in the other patients (Figure 6B and Table S6).

## 4. Discussion

Clinical outcomes of patients with MDS who experience HMA treatment failure are poor [34,35]. Thus, elucidating the resistance mechanism is important for overcoming this problem. Drug-resistant cell line models can be useful in vitro tools for understanding the mechanisms underlying clinical anticancer drug resistance, enabling the discovery of molecular alterations between a drug-resistant cell line and its drug-sensitive counterpart. Furthermore, acquired-resistance cell line models can play an important additional role in revealing the mechanisms of action of new anticancer drugs [36]. It has also been shown that the expression levels of certain cancer-related genes can be altered in DEC-resistant cell lines and that the regulation of gene expression levels can influence resistance [37]. To date, most research on DEC resistance has focused on resistant cells derived from a few cell lines originating from acute promyelocytic leukemia (HL-60) or chronic myeloid leukemia (K562 cells) [11,13,37]. However, DEC is not used to treat acute promyelocytic leukemia or chronic myeloid leukemia. Accordingly, data obtained from HL-60 or K562 cells cannot be expected to accurately represent the phenomenon of DEC resistance in actual MDS patients.

The DEC-resistant MDS cell line developed for this study was produced by continuously exposing F-36P cells to staged increases in DEC concentration. DEGs associated with DEC resistance were determined by biological and bioinformatic analyses of the gene expression profiles of parental F-36P and F-36P/DEC cells. On the basis of this expression profiling, we selected 38 candidate DEGs, including those involved in immune cell activation, the regulation of myeloid cell differentiation, transcriptional misregulation, PI3K-Akt signaling, and the JAK-STAT signaling pathway, for further analysis. These analyses identified pathways that overlap with previously reported pathways; however, the individual genes were quite different.

A number of genetic studies performed to date have identified groups of mutated genes that contribute to the pathogenesis of MDS and resistance to HMAs [20,38]. These genes were categorized according to a limited number of cellular processes, including epigenetic, RNA splicing and traditional transcriptional regulation, and signal transduction. Several large studies have evaluated the prognostic value of MDS-associated gene mutations across a broad cross-section of patients [39,40,41]. Somatic mutations in certain genes reproducibly predict patient prognosis. Across studies, mutations in genes encoding ETV6, RUNX1, TP53, EZH2, ASXL1, FLT3-ITD, and SRSF2 predict poor overall survival, whereas mutations in SF3B1 are associated with better clinical outcomes [40,42]. It is also known that mutations in TP53 and TET2 affect the prognosis of patients undergoing DEC therapy [17]. Considering the high incidence of mutations, it can be assumed that mutations play a role in MDS pathogenesis and resistance to HMAs. However, many such studies did not distinguish specific mutations that were closely related to an incomplete response to treatment. Therefore, it is reasonable to suggest that the decision to treat MDS patients with HMAs should not be based solely on the basis of mutation information.

We next looked for possible regulatory mechanisms governing the expression of these genes, focusing on FOS, a component of transcription factor AP-1 (activator protein 1), and PI3K-AKT signaling pathways, which play essential roles in hematological malignancies. We found that FOS transcriptionally induced the expression of genes encoding the co-inhibitory immune checkpoint proteins PD-1 (programmed cell death protein-1) and PD-L1 (ligand for PD-1) through binding to enhancer regions of the respective gene promoters. It has additionally been shown that blockade of the FOS-mediated induction of PD-1 could be harnessed therapeutically to restore T cell-mediated antitumor responses [43]. Impairment of AKT3, a member of the AKT protein family, has also been linked to multiple myeloma, and mutations in the AKT3 gene have been frequently reported in de novo Philadelphia chromosome-positive AML. Accordingly, the presence of a mutated AKT3 gene is correlated with, and predicts, a poor clinical response in MDS patients [44]. Interestingly, another study confirmed that AKT3 protein levels are overexpressed in the plasma of MDS patients [45]. In DEC-induced DNA reprogramming, chimeric antigen receptor T (CAR T) cells, compared to the control group, differentially expressed genes including AKT3, and it is expected that this can enhance antitumor effects or reduce tumor recurrence [46]. We found that AKT3 and FOS genes were overexpressed in F36P/DEC cells, and clinical data even showed overexpression of these genes in MDS patients with poor prognosis.

In the current study, a bioinformatics analysis was used to identify biological networks, and in vitro experiments were conducted to verify bioinformatics findings. Our examination of the roles of certain molecules and the demonstration of the involvement of specific signaling pathways in the current study is expected to expand our understanding of the molecular mechanism of DEC resistance. However, our study is not without limitations, including the need to validate our findings in a larger group of patients and to perform in-depth experiments to confirm that the tendency toward the increased expression of genes identified in vitro is recapitulated in vivo. Future advances should yield more accurate data from bioinformatics analyses, confirming pathways that are significant for DEC resistance in MDS and potentially achieving a comprehensive understanding of this process.

In conclusion, we successfully established a good in vitro model with potential utility in understanding the molecular mechanisms and origin of DEC resistance. Using this model, we observed the increased expression of select genes from among 38 DEGs identified, including AKT3 and FOS, in F-36P/DEC cells and showed that they were associated with several pathways with potential involvement in DEC resistance, consistent with the results of our bioinformatics analysis. Our findings can serve as a reference for other researchers involved in investigating the development of resistance to DEC and provide tools that could aid in the application of combination therapeutics and biomarkers as therapies for MDS patients.

## Figures and Tables

**Table 1:** GO analysis of DEC resistance-related DEGs.

Category	Term	Description	Count	*p*-Value
BP	GO:0045893	Regulation of transcription, DNA-templated	15	5.89 × 10^−7^
	GO:0045944	Regulation of transcription from RNA polymerase II promoter	18	1.01 × 10^−5^
	GO:0008285	Negative regulation of cell proliferation	9	3.59 × 10^−5^
	GO:0043066	Negative regulation of apoptotic process	9	9.52 × 10^−5^
	GO:0007165	Signal transduction	12	1.00 × 10^−3^
	GO:0007411	Axon guidance	5	0.0017
	GO:0007050	Cell cycle arrest	4	0.0105
	GO:0007568	Aging	4	0.0160
	GO:0016055	Wnt signaling pathway	4	0.0223
	GO:0071425	Hematopoietic stem cell proliferation	2	0.0441
CC	GO:0005654	Nucleoplasm	25	0.0000
	GO:0005634	Nucleus	29	0.0005
	GO:0008305	Integrin complex	3	0.0029
	GO:0009925	Basal plasma membrane	3	0.0036
	GO:0009986	Cell surface	7	0.0052
	GO:0005829	Cytosol	19	0.0055
	GO:0005576	Extracellular region	12	0.0068
	GO:0005886	Plasma membrane	20	0.0241
	GO:0005925	Focal adhesion	5	0.0285
	GO:0005737	Cytoplasm	23	0.0377
MF	GO:0005515	Protein binding	46	0.0000
	GO:0003700	Transcription factor activity, sequence-specific DNA binding	13	0.0000
	GO:0008134	Transcription factor binding	6	0.0021
	GO:0001205	Transcriptional activator activity, RNA polymerase II distal enhancer sequence-specific binding	3	0.0029
	GO:0003682	Chromatin binding	6	0.0081
	GO:0003677	DNA binding	12	0.0155
	GO:0046982	Protein heterodimerization activity	6	0.0162
	GO:0019903	Protein phosphatase binding	3	0.0173
	GO:0004672	Protein kinase activity	5	0.0276
	GO:0019901	Protein kinase binding	5	0.0320

**Table 2:** Clinical characteristics of the MDS patients treated with DEC.

Sample No.	1	2	3	4
Sex	Female	Male	Male	Female
Age (years)	46	73	74	61
Weight (kg)	60	66	76	55
Height (m)	1.56	1.6	1.63	1.47
BMI (kg/m^2^)	24.7	25.8	28.6	25.5
Underlying disease	History of allogeneic HSCT due to aplastic anemia, paroxysmal nocturnal hemoglobinuria	Diabetes mellitus	Diabetes mellitus	None
Baseline clinical characteristics				
WBC (×10^6^/L)	3370	6700	2970	3490
ANC (×10^6^/L)	135	1100	535	733
Hb (g/dL)	9.7	8.7	7	10.4
Platelets (×10^9^/L)	11	37	32	200
BM blasts (%)	10.3	20	6	12.5
Cytogenetic abnormalities	Complex karyotype ^1^	None	Complex karyotype ^2^	del (20q)
IPSS	3.0	2.0	2.0	1.5
IPSS risk category	High	Int-2	Int-2	Int-2
IPSS-R	9.5	6.0	9.0	4.5
IPSS-R risk category	Very high	High	Very high	Int
MDS subtypes (WHO)	MDS-EB2	MDS-EB2	MDS-EB1	MDS-EB2
Treatment cycle of DEC	2	16	11	5
Best response	CR	PR	CR	CR
Progression (Leukemic transformation)	Yes	Yes	Yes	Yes
PFS (month)	9	46	11	24
Allogeneic HSCT	Yes	No	No	Yes
Time to HSCT (month)	4	None	None	7
F/U period (month)	11	52	11	24
F/U result	Dead	Dead	Dead	Dead
Cause of death	Leukemia	Leukemia	Leukemia	Pneumonia

Alterations were found in 0% to 0.8% of the respective analyses and are depicted graphically. Many candidate genes exhibited amplification alterations. Abbreviations: BMI, Body mass index; WBC, white blood cell count; ANC, absolute neutrophil count; Hb, hemoglobin; BM, bone marrow, IPSS, International Prognostic Scoring System; IPSS-R, Revised International Prognostic Scoring System; Int, intermediate; MDS-EB, MDS with excess blasts; CR, complete remission; PR, partial remission; PFS, progression-free survival; HSCT, hematopoietic stem cell transplantation; F/U, follow-Up. ^1^ 41X, -Y, add(5)(q11.2), der(7;17)(p10;q10), -16, -18, -20, add(22)(q13), dup(1)(q12q42), add(4)(p14), add(4)(q21), add(6)(q13), add(7)(p13), -8, -add(12), +12, -13. ^2^ 44~45XY, add(1)(p32), -5, i(14)(q10), -17, 18, -21, +add(22)(q13)×2, +mar1, +mar2.

## Data Availability

Data sharing not applicable.

## Supplementary Materials

The following are available online at https://www.mdpi.com/article/10.3390/cells10123494/s1, Table S1: qRT-PCR primer sequences for the verification of candidate DEGs. Table S2: A cluster heatmap showing 189 differentially expressed genes. Table S3: The 30 most differentially expressed genes from the volcano plot. Table S4: DEGs between F-36P and F-36P/DEC cells. Table S5: Functional roles of module genes. Table S6: Expression levels of 38 genes in bone marrow from MDS patients.
