# Supplementary material for: Gene Expression Profiles Identify Biomarkers of Resistance to Decitabine in Myelodysplastic Syndromes
Source: Cells. 2021 Dec 10;10(12):3494. doi: 10.3390/cells10123494 (PMC8700444; doi:10.3390/cells10123494)
Supplement: Supplementary file 1 [file cells-10-03494-s001.zip › Table S1_primer 38.pdf]

Table S1. qRT-PCR primer sequences for the verification of candidate DEGs.

Gene name	Primer Sequences	Accession No.	Product size
AKT3	(F) TCCAGGCTGTAGCAGACAGA<br>(R) AAAACAGCTCGCCCCCATTA	NM_181690	399
AMER1	(F) CCCACACTTGGGAAGCTCAT<br>(R) CACAGTGGCATTCCCTTCCT	NM_152424	464
BCL2	(F) AGAGGGGCTACGAGTGGGAT<br>(R) TTGACGCTCTCCACACACAT	NM_000657	415
BCL2A1	(F) CTGCAGTGCGTCCTACAGAT<br>(R) GTATCCACATCCGGGGCAAT	NM_004049	293
CDKN1A	(F) GCGACTGTGATGCGCTAATG<br>(R) ATCTGTCATGCTGGTCTGCC	NM_000389	353
CDKN2A	(F) ACCGAATAGTTACGGTCGGAG<br>(R) CCTGCTTCTACAAACCCACAA	NM_001195132	376
CDKN2D	(F) CGCTGCAGGTCATGATGTTT<br>(R) CGTGCTCCACTAGGACCTTC	NM_001800	156
CR1	(F) CCCACATCCACCCAAGATCC<br>(R) TTGTTTGCAGAGTTCGGGGA	NM_000573	548
DLC1	(F) ACGCAATTCTTCCAGCTCCA<br>(R) TCGTTGTCCACATCCAGGT	NM_001348081	276
DNMT1	(F) ATGAGCAGCCCATCTTCCTG<br>(R) CCTCATCGTCATCTGCCTCC	BC126227	399
EGF	(F) CCTGAAGGCTCAGTGCTTGA<br>(R) CTTCAGGGCTGTATGGGCAA	NM_001963	333
ETS1	(F) TCCCCTCCCCGGGTAAACTC<br>(R) CCTTATTGAGGTCAGCACGGT	NM_001162422	231
FGF11	(F) CCTCATCCTGCTGTCCAAGG<br>(R) AGCGACACTCAGCTGTGAAA	NM_004112	305
FOS	(F) TACTACCACTCACCCGCAGA<br>(R) CAGGTTGGCAATCTCGGTCT	NM_005252	474
GFI1	(F) GAACGGAGCTCGGAGTTTGA<br>(R) CCACCTTCCTCTGGAAACCC	NM_005263	988
GNA11	(F) AGTGCCATCAAGACCCTGTG<br>(R) TCTTCAGGATGAACTCCCGC	NM_002067	547
HSPA2	(F) GAGCTCAATGCCGACCTCTT<br>(R) AGTACGCTGCTCTGGTTGTC	NM_021979	407
ID1	(F) CTGGACGAGCAGCAGGTAAA<br>(R) TCAGCGACACAAGATGCGAT	NM_002165	300
ID2	(F) TGAAAGCCTTCAGTCCCGTG<br>(R) TGGTGATGCAGGCTGACAAT	NM_002166	271
IL12B	(F) GTCACAAAGGAGGCGAGGTT<br>(R) CAGCAGGTGAAACGTCCAGA	NM_002187	160
ITGA9	(F) GACCGCGATGATGAGTGGAT<br>(R) CATCAATGGTGGACGGGTGA	NM_002207	436
KL	(F) GTGGCCGAGAGAGTTTTGGA<br>(R) GGGAGGTCTCCGTACTTGAA	AB005141	359
KMT2E	(F) TCCTGGAGAAAAGGAACCTGC<br>(R) GTTTCCATAAACATGCCTCTGGTGT	AF519459	318

LIG4	(F)<br>(R)	TGTGCAGTAGCAGAGAAGCC<br>TGTTCTAGGTCGTCCAGGGT	NM_002312	350
LMX1B	(F)<br>(R)	AGAGACACTGGCAGCTGA<br>TCGTTCCCTGGCATTTGGG	NM_002316	291
MAPK10	(F)<br>(R)	TTCACACCCCAGAAAACGCT<br>CTTGGGCCGATTCTCCACAT	NM_002753	522
MET	(F)<br>(R)	CGCACAAAGCAAGCCAGATT<br>GAGGCATTGACTGCAGGACT	NM_000245	553
MLF1	(F)<br>(R)	TCCATTCTTGCACACCGAGA<br>TGGAGCTCGACGAGTTTGAG	NM_022443	351
NFKB1	(F)<br>(R)	GGGCACTGGAAGTACAGGTC<br>TCCTGCACAGCAGTGAGATG	NM_003998.2	435
NOTCH1	(F)<br>(R)	TGCACACTATTCTGCCCCAG<br>ACTTGAAGGCCTCCGGAATG	NM_017617	309
PRKAA2	(F)<br>(R)	TCTTCTTTGTGGCACCCTCC<br>ATAAGCCACTGCAAGCTGGT	NM_006252	355
RELN	(F)<br>(R)	TGGCTATTACCCCCGCTTTT<br>ACGTGAGAGGCTACCACACT	NM_005045.2	318
RUNX1T1	(F)<br>(R)	TGCAGCAGTTTGGCAATGAC<br>TGGTTCTGTCTGGAGTTCGC	NM_004349	348
SHH	(F)<br>(R)	TCCACTGCTCGGTGAAAGCAG<br>AGCTGTGCTCCTCGATGACC	NM_000193	674
SPP1	(F)<br>(R)	GAATCTCCTAGCCCCACAGACC<br>TGTGAGGTGATGTCCTCGTCT	NM_000582	378
STAT3	(F)<br>(R)	TCAAGAGTCAAGGAGACATG<br>AGTCTTTGTCAATGCACACT	NM_213662	579
TNFRSF10B	(F)<br>(R)	AACAAAAGAGGTCCAGCCCC<br>TCCCCACTGTGCTTTGTACC	NM_003842.3	361
TUBB3	(F)<br>(R)	TCACTACACGGAGGGGGCGG<br>CGGGAAGCGCAAGGAGGTGG	NM_006086.3	418
GAPDH	(F)<br>(R)	AAGGACTCATGACCACAGTC<br>TTCAGCTCAGGGATGACCTT	NM_001289745.2	160
