# Supplementary material for: Gene Expression Profiles Identify Biomarkers of Resistance to Decitabine in Myelodysplastic Syndromes
Source: Cells. 2021 Dec 10;10(12):3494. doi: 10.3390/cells10123494 (PMC8700444; doi:10.3390/cells10123494)
Supplement: Supplementary file 1 [file cells-10-03494-s001.zip › Table S2_189 genes.pdf]

Table S2. 189 differentially expressed genes from a cluster heatmap.

No.	Gene Name	Description
1	RELN	Reelin
2	CACNA2D1	Calcium channel, voltage-dependent, alpha 2/delta subunit 1
3	SMC1B	Structural maintenance of chromosomes protein 1B
4	PTPRR	Protein tyrosine phosphatase, receptor type, R
5	TNFRSF10B	Tumor necrosis factor receptor superfamily, member 10b
6	IL2RB	High affinity IL-2 receptor subunit beta
7	MAPK8IP2	Mitogen-activated protein kinase 8 interacting protein 2
8	CRLF2	Thymic stromal lymphopoietin protein receptor
9	SFRP2	Secreted apoptosis-related protein 1
10	MET	MET proto-oncogene, receptor tyrosine kinase
11	ITGB8	Integrin, beta 8
12	HMGA2	High mobility group AT-hook protein 2
13	MAPK8IP1	Mitogen-activated protein kinase 8 interacting protein 1
14	MECOM	Ecotropic virus integration site 1 protein homolog
15	FZD10	Frizzled class receptor 10
16	GATA3	Trans-acting T-cell-specific transcription factor GATA-3
17	LEFTY2	Endometrial bleeding-associated factor
18	BCL2A1	Hemopoietic-specific early response protein
19	MMP9	Matrix metallopeptidase 9 (gelatinase B, 92kDa gelatinase, 92kDa type IV collagenase)
20	CACNG6	Neuronal voltage-gated calcium channel gamma-6 subunit
21	CACNA2D2	Calcium channel, voltage-dependent, alpha 2/delta subunit 2
22	IL1R2	CD121 antigen-like family member B
23	GLI3	Transcriptional activator GLI3
24	HES1	Class B basic helix-loop-helix protein 39
25	PLA2G4C	Phospholipase A2, group IVC (cytosolic, calcium-independent)
26	GRIA3	Glutamate receptor, ionotropic, AMPA 3
27	PRL	Prolactin
28	SETBP1	SET binding protein 1
29	AKT3	V-akt murine thymoma viral oncogene homolog 3
30	CCR7	Epstein-Barr virus-induced G-protein coupled receptor 1
31	LRP2	Low density lipoprotein receptor-related protein 2
32	COL24A1	Collagen alpha-1(XXIV) chain
33	RASGRF2	Ras protein-specific guanine nucleotide-releasing factor 2
34	RNF43	RING-type E3 ubiquitin transferase RNF43
35	TSHR	Thyroid stimulating hormone receptor
36	WIF1	WNT inhibitory factor 1
37	PPP3R2	Protein phosphatase 3 regulatory subunit B beta isoform
38	MPO	Myeloperoxidase
39	NTF3	Nerve growth factor 2
40	PROM1	Prominin-like protein 1
41	DTX1	RING-type E3 ubiquitin transferase DTX1

42	NTRK2	Neurotrophic tyrosine kinase, receptor, type 2
43	BCL2	Apoptosis regulator Bcl-2
44	DDIT4	Protein regulated in development and DNA damage response 1
45	NODAL	Nodal growth differentiation factor
46	IL20RA	Interleukin-20 receptor subunit alpha
47	IL12B	Cytotoxic lymphocyte maturation factor 40 kDa subunit
48	IGFBP3	Insulin-like growth factor binding protein 3
49	ETS1	V-ets avian erythroblastosis virus E26 oncogene homolog 1
50	INHBA	Erythroid differentiation protein
51	SFN	Epithelial cell marker protein 1
52	HSPA2	Heat shock-related 70 kDa protein 2
53	LAMB4	Laminin beta-1-related protein
54	LAMC3	Laminin-12 subunit gamma
55	KMT2E	Myeloid/lymphoid or mixed-lineage leukemia protein 5
56	NGFR	Tumor necrosis factor receptor superfamily member 16
57	TCF7L1	Transcription factor 7-like 1 (T-cell specific, HMG-box)
58	NOTCH1	Translocation-associated notch protein TAN-1
59	PDGFB	Platelet-derived growth factor beta polypeptide
60	KL	Klotho
61	TPO	Thyroid peroxidase
62	ID2	Inhibitor of DNA binding 2, dominant negative helix-loop-helix protein
63	FGF23	Tumor-derived hypophosphatemia-inducing factor
64	DLC1	StAR-related lipid transfer protein 12
65	HPGD	Short chain dehydrogenase/reductase family 36C member 1
66	CR1	Complement component (3b/4b) receptor 1 (Knops blood group)
67	LTBP1	Latent transforming growth factor beta binding protein 1
68	RUNX1T1	Runt-related transcription factor 1
69	NOTCH3	Neurogenic locus notch homolog protein 3
70	TUBB3	Tubulin, beta 3 class III
71	IRS1	Insulin receptor substrate 1
72	SHH	Shh unprocessed N-terminal signaling and C-terminal autoprocessing domains
73	DKK2	Dickkopf WNT signaling pathway inhibitor 2
74	CDKN1A	Cyclin-dependent kinase inhibitor 1A (p21, Cip1)
75	TNN	Tenascin N
76	AMER1	Wilms tumor gene on the X chromosome protein
77	SSX1	Synovial sarcoma, X breakpoint 1
78	LMX1B	LIM homeobox transcription factor 1, beta
79	COL27A1	Collagen alpha-1(XXVII) chain
80	PITX2	RIEG bicoid-related homeobox transcription factor
81	GFI1	Growth factor independent 1 transcription repressor
82	PRMT8	Heterogeneous nuclear ribonucleoprotein methyltransferase-like protein 4
83	MAPT	Microtubule-associated protein tau

84	LAMA1	Laminin subunit alpha-1
85	CALML3	Calmodulin-related protein NB-1
86	WNT6	Wingless-type MMTV integration site family, member 6
87	EGF	Pro-epidermal growth factor
88	CEBPE	CCAAT/enhancer binding protein (C/EBP), epsilon
89	FGF21	Fibroblast growth factor 21
90	SMAD3	Mothers against decapentaplegic homolog 3
91	WNT2	Wingless-type MMTV integration site family member 2
92	APH1B	Presenilin-stabilization factor-like
93	GDF6	Growth/differentiation factor 16
94	SMAD9	Mothers against decapentaplegic homolog 9
95	CASP7	Caspase 7, apoptosis-related cysteine peptidase
96	COL4A4	Collagen alpha-4(IV) chain
97	PPP2R2B	Serine/threonine-protein phosphatase 2A 55 kDa regulatory subunit B beta isoform
98	FGF3	Heparin-binding growth factor 3
99	PLAT	Tissue-type plasminogen activator
100	IL1RAP	Interleukin 1 receptor accessory protein
101	CALML5	Calmodulin-like skin protein
102	CACNA1C	Calcium channel, L type, alpha-1 polypeptide, isoform 1, cardiac muscle
103	CCNB3	G2/mitotic-specific cyclin-B3
104	FN1	Fibronectin type III domain containing
105	IL12A	Cytotoxic lymphocyte maturation factor 35 kDa subunit
106	FLT1	Vascular endothelial growth factor receptor 1
107	IL6	B-cell stimulatory factor 2
108	ETV7	ETS translocation variant 7
109	IFNA7	Interferon alpha-J1
110	NUMBL	Numb homolog (Drosophila)-like
111	CDKN2A	Cyclin-dependent kinase 4 inhibitor A
112	FGFR4	Fibroblast growth factor receptor 4
113	ITGB7	Gut homing receptor beta subunit
114	PGF	Placental growth factor
115	TLX1	T-cell leukemia homeobox protein 1
116	CCNO	Cyclin O
117	HSPA6	Heat shock 70kDa protein 6 (HSP70B')
118	IL13RA2	Interleukin-13 receptor subunit alpha-2
119	ITGB4	Integrin, beta 4
120	PLA2G3	Phosphatidylcholine 2-acylhydrolase 3
121	IL1B	Interleukin 1, beta
122	COL11A1	Collagen alpha-1(XI) chain
123	PLCE1	1-phosphatidylinositol 4,5-bisphosphate phosphodiesterase epsilon-1
124	COMP	Cartilage oligomeric matrix protein
125	MAPK10	Stress-activated protein kinase JNK3
126	WNT10A	Wingless-type MMTV integration site family, member 10A

127	WNT3	Wingless-type MMTV integration site family, member 3
128	CLCF1	Cardiotrophin-like cytokine factor 1
129	CNTFR	Ciliary neurotrophic factor receptor subunit alpha
130	SOX17	SRY (sex determining region Y)-box 17
131	RASAL1	RAS protein activator like 1 (GAP1 like)
132	IL15	Interleukin 15
133	CDKN2B	Cyclin-dependent kinase inhibitor 2B (p15, inhibits CDK4)
134	MLF1	Myelodysplasia-myeloid leukemia factor 1
135	COL3A1	Collagen alpha-1(III) chain
136	FZD9	Frizzled class receptor 9
137	CDH1	Cadherin 1, type 1, E-cadherin (epithelial)
138	IGF1	Insulin-like growth factor 1 (somatomedin C)
139	FGF20	Fibroblast growth factor 20
140	PDGFRB	Platelet-derived growth factor receptor, beta polypeptide
141	ZBTB32	Zinc finger and BTB domain-containing protein 32
142	FLNC	Actin-binding-like protein
143	PLA2G4A	Phospholipase A2, group IVA (cytosolic, calcium-dependent)
144	CREB3L3	Cyclic AMP-responsive element-binding protein 3-like protein 3
145	IL3RA	Interleukin 3 receptor, alpha (low affinity)
146	EPHA2	Tyrosine-protein kinase receptor ECK
147	RAC3	Ras-related C3 botulinum toxin substrate 3 (rho family, small GTP binding protein Rac3)
148	DDIT3	Growth arrest and DNA damage-inducible protein GADD153
149	IL22RA1	Interleukin-22 receptor subunit alpha-1
150	CXXC4	Inhibition of the Dvl and axin complex protein
151	MPL	MPL proto-oncogene, thrombopoietin receptor
152	STAT3	Signal transducer and activator of transcription 3 (acute-phase response factor)
153	TLR2	Toll/interleukin-1 receptor-like protein 4
154	MMP7	Matrix metallopeptidase 7 (matrilysin, uterine)
155	WNT2B	Wingless-type MMTV integration site family, member 2B
156	MMP3	Matrix metallopeptidase 3 (stromelysin 1, progelatinase)
157	ETV1	ETS translocation variant 1
158	CACNA1H	Calcium channel, voltage-dependent, T type, alpha 1H subunit
159	GNA11	Guanine nucleotide binding protein (G protein), alpha 11 (Gq class)
160	PPARGC1A	Peroxisome proliferator-activated receptor gamma, coactivator 1 alpha
161	IFNA17	Interferon, alpha 17
162	ID1	Inhibitor of DNA binding 1, dominant negative helix-loop-helix protein
163	B2M	Beta-2-microglobulin
164	PRKAA2	5'-AMP-activated protein kinase catalytic subunit alpha-2
165	FGF16	Fibroblast growth factor 16
166	FGF1	Fibroblast growth factor 1 (acidic)
167	HDAC11	Histone deacetylase 11
168	NFKBIZ	Nuclear factor of kappa light polypeptide gene enhancer in B-cells inhibitor, zeta

169	NOG	Noggin
170	CSF3R	Colony stimulating factor 3 receptor (granulocyte)
171	SPP1	Secreted phosphoprotein 1
172	DLL3	Drosophila Delta homolog 3
173	CHEK2	Serine/threonine-protein kinase Chk2
174	CALML6	Calglandulin-like protein
175	CDKN2D	Cyclin-dependent kinase inhibitor 2D (p19, inhibits CDK4)
176	EIF4EBP1	Phosphorylated heat- and acid-stable protein regulated by insulin 1
177	DNMT1	DNA (cytosine-5-)-methyltransferase 1
178	FGF8	Fibroblast growth factor 8 (androgen-induced)
179	LEPR	Leptin receptor
180	ITGA9	Integrin alpha-RLC
181	FLNA	Endothelial actin-binding protein
182	COL4A5	Collagen alpha-5(IV) chain
183	FGF11	Fibroblast growth factor homologous factor 3
184	WNT5A	Wingless-type MMTV integration site family, member 5A
185	RASA4	Calcium-promoted Ras inactivator
186	MAD2L2	Mitotic spindle assembly checkpoint protein MAD2B
187	FOS	FBJ murine osteosarcoma viral oncogene homolog
188	NFKB1	Nuclear factor of kappa light polypeptide gene enhancer in B-cells 1
189	LIG4	Polydeoxyribonucleotide synthase [ATP] 4
