# Supplementary material for: Gene Expression Profiles Identify Biomarkers of Resistance to Decitabine in Myelodysplastic Syndromes
Source: Cells. 2021 Dec 10;10(12):3494. doi: 10.3390/cells10123494 (PMC8700444; doi:10.3390/cells10123494)
Supplement: Supplementary file 1 [file cells-10-03494-s001.zip › Table S3_30 genes.pdf]

Table S3. The 30 most differentially expressed genes from the volcano plot.

No.	Gene Name	Log_2 fold change	P-value	Gene sets
1	RELN	3.86	0.000	PI3K
2	MET	2.27	0.015	Driver Gene, PI3K, Ras, Transcriptional Misregulation
3	PTPRR	2.25	0.005	MAPK
4	PLA2G4C	1.98	0.009	MAPK, Ras
5	MECOM	1.93	0.018	MAPK
6	BCL2A1	1.61	0.037	Transcriptional Misregulation
7	HES1	1.46	0.005	Notch
8	AKT3	1.40	0.003	Cell Cycle - Apoptosis, JAK-STAT, MAPK, PI3K, Ras
9	HSPA6	1.30	0.036	MAPK
10	CCNB3	1.21	0.003	Cell Cycle - Apoptosis
11	DDIT4	1.21	0.011	PI3K
12	LTBP1	1.14	0.025	TGF-beta
13	WNT6	1.13	0.041	Hedgehog, Wnt
14	EGF	1.09	0.032	MAPK, PI3K, Ras
15	SMAD3	1.01	0.003	Cell Cycle - Apoptosis, TGF-beta, Wnt
16	CASP7	1.01	0.014	Cell Cycle - Apoptosis
17	IL1RAP	1.00	0.032	Cell Cycle - Apoptosis
18	DUSP5	-1.01	0.022	MAPK
19	ITGA6	-1.03	0.006	PI3K
20	CACNB4	-1.19	0.028	MAPK
21	FGFR3	-1.32	0.006	Driver Gene, MAPK, PI3K, Ras
22	LAMA5	-1.33	0.005	PI3K
23	SPP1	-1.36	0.015	PI3K
24	PPP2R2C	-1.44	0.001	PI3K
25	CEBPA	-1.53	0.003	Driver Gene, Transcriptional Misregulation
26	IL8	-1.63	0.002	Transcriptional Misregulation
27	THBS1	-1.83	0.002	PI3K, TGF-beta
28	TNFRSF10D	-1.88	0.012	Cell Cycle - Apoptosis
29	BDNF	-2.06	0.004	MAPK
30	FST	-2.42	0.000	TGF-beta

All expression were performed in triplicate, and value are significant at the $p < 0.05$ level.
