# Supplementary material for: Gene Expression Profiles Identify Biomarkers of Resistance to Decitabine in Myelodysplastic Syndromes
Source: Cells. 2021 Dec 10;10(12):3494. doi: 10.3390/cells10123494 (PMC8700444; doi:10.3390/cells10123494)
Supplement: Supplementary file 1 [file cells-10-03494-s001.zip › Table S4_gene string 54.pdf]

Table S4. DEGs between F-36P and F-36P/DEC cells.

	Gene Name	Accession Number	Description
1	AKT3	NM_181690	V-akt murine thymoma viral oncogene homolog 3
2	AMER1	NM_152424	Wilms tumor gene on the X chromosome protein
3	BCL2	NM_000657	Apoptosis regulator Bcl-2
4	BCL2A1	NM_004049	Hemopoietic-specific early response protein
5	BCL2L11	NM_207002	Bcl2-interacting mediator of cell death
6	CDKN1A	NM_000389	Cyclin-dependent kinase inhibitor 1A
7	CDKN2A	NM_001195132	Cyclin-dependent kinase 4 inhibitor A
8	CDKN2D	NM_001800	Cyclin-dependent kinase inhibitor 2D
9	CREB3L4	NM_130898	Cyclic AMP-responsive element-binding protein 3-like protein 4
10	DUSP6	BC_037236	Mitogen-activated protein kinase phosphatase 3
11	EGF	NM_001963	Pro-epidermal growth factor
12	EPO	NM_000799.2	Erythropoietin
13	ETS1	NM_001162422	V-ets avian erythroblastosis virus E26 oncogene homolog 1
14	FGF1	NM_033137.1	Fibroblast growth factor 1
15	FGF11	NM_004112	Fibroblast growth factor homologous factor 3
16	FOS	NM_005252	FBJ murine osteosarcoma viral oncogene homolog
17	FZD7	NM_003507	Frizzled class receptor 7
18	GFI1	NM_005263	Growth factor independent 1 transcription repressor
19	GNA11	NM_002067	Guanine nucleotide binding protein, alpha 11
20	HMGA1	NM_145904	High mobility group protein HMG-I/HMG-Y
21	HSPA2	NM_021979	Heat shock-related 70 kDa protein 2
22	ID1	NM_002165	Inhibitor of DNA binding 1, dominant negative helix-loop-helix protein
23	ID2	NM_002166	Inhibitor of DNA binding 2, dominant negative helix-loop-helix protein
24	IFNA17	NM_021268	Interferon, alpha 17
25	IL11RA	NM_147162	Interleukin-11 receptor subunit alpha
26	IL12B	NM_002187	Cytotoxic lymphocyte maturation factor 40 kDa subunit
27	ITGA1	NM_181501	CD49 antigen-like family member A
28	ITGA6	NM_000210	CD49 antigen-like family member F
29	ITGA9	NM_002207	Integrin alpha-RLC
30	KL	AB_005141	Klotho
31	KMT2E	AF_519459	Myeloid/lymphoid or mixed-lineage leukemia protein 5
32	LMX1B	NM_002316	LIM homeobox transcription factor 1, beta

33	LTBP1	NM_000627	Latent transforming growth factor beta binding protein 1
34	MAPK10	NM_002753	Stress-activated protein kinase JNK3
35	MCL1	AF_118124	Induced myeloid leukemia cell differentiation protein Mcl1
36	MECOM	NM_005241	Ecotropic virus integration site 1 protein homolog
37	MET	NM_000245	MET proto-oncogene, receptor tyrosine kinase
38	NFATC1	NM_172389	Nuclear factor of activated T-cells, cytoplasmic, calcineurin-dependent 1
39	NFKB1	NM_003998.2	Nuclear factor of kappa light polypeptide gene enhancer in B-cells 1
40	NFKBIZ	NM_001005474	Nuclear factor of kappa light polypeptide gene enhancer in B-cells inhibitor, zeta
41	NOTCH1	NM_017617	Translocation-associated notch protein TAN-1
42	PRKAA2	NM_006252	5'-AMP-activated protein kinase catalytic subunit alpha-2
43	PTPRR	NM_001207015	Protein tyrosine phosphatase, receptor type, R
44	RELN	NM_005045.2	Reelin
45	RPS6KA5	NM_004755	Nuclear mitogen- and stress-activated protein kinase 1
46	RUNX1T1	NM_004349	Runt-related transcription factor 1
47	SHH	NM_000193	Shh unprocessed N-terminal signaling and C-terminal autoprocessing domains
48	SPP1	NM_000582	Secreted phosphoprotein 1
49	SPRY2	NM_005842	Sprouty homolog 2
50	STAT3	NM_213662	Signal transducer and activator of transcription 3
51	TNFRSF10B	NM_003842.3	Tumor necrosis factor receptor superfamily, member 10b
52	TUBB3	NM_006086.3	Tubulin, beta 3 class III
53	WNT3	NM_030753	Wingless-type MMTV integration site family, member 3
54	WNT5A	NM_003392	Wingless-type MMTV integration site family, member 5A
