# Supplementary material for: Gene Expression Profiles Identify Biomarkers of Resistance to Decitabine in Myelodysplastic Syndromes
Source: Cells. 2021 Dec 10;10(12):3494. doi: 10.3390/cells10123494 (PMC8700444; doi:10.3390/cells10123494)
Supplement: Supplementary file 1 [file cells-10-03494-s001.zip › Table S5_function 38.pdf]

Table S5. Functional roles of module genes.

Gene name	Identifier	Description
AKT3	ENSP000 00263826	V-akt murine thymoma viral oncogene homolog 3; AKT3 is one of 3 closely related serine/threonine- protein kinases (AKT1, AKT2 and AKT3) called the AKT kinase, and which regulate many processes including metabolism, proliferation, cell survival, growth and angiogenesis. This is mediated through serine and/or threonine phosphorylation of a range of downstream substrates. Over 100 substrate candidates have been reported so far, but for most of them, no isoform specificity has been reported. AKT3 is the least studied AKT isoform. It plays an important role in brain development and is crucial for the viability of malignant glioma cells. AKT3 isoform may also be the key molecule in up-regulation and down-regulation of MMP13 via IL13. Required for the coordination of mitochondrial biogenesis with growth factor-induced increases in cellular energy demands. Down-regulation by RNA interference reduces the expression of the phosphorylated form of BAD, resulting in the induction of caspase- dependent apoptosis; Pleckstrin homology domain containing
AMER1	ENSP000 00329117	Wilms tumor gene on the X chromosome protein; Regulator of the canonical Wnt signaling pathway. Acts by specifically binding phosphatidylinositol 4,5-bisphosphate (PtdIns(4,5)P2), translocating to the cell membrane and interacting with key regulators of the canonical Wnt signaling pathway, such as components of the beta-catenin destruction complex. Acts both as a positive and negative regulator of the Wnt signaling pathway, depending on the context: acts as a positive regulator by promoting LRP6 phosphorylation. Also acts as a negative regulator by acting as a scaffold protein for the beta- catenin destruction complex and promoting stabilization of Axin at the cell membrane. Promotes CTNNB1 ubiquitination and degradation. Involved in kidney development; Belongs to the Amer family.
BCL2	ENSP000 00381185	Apoptosis regulator Bcl-2; Suppresses apoptosis in a variety of cell systems including factor-dependent lymphohematopoietic and neural cells. Regulates cell death by controlling the mitochondrial membrane permeability. Appears to function in a feedback loop system with caspases. Inhibits caspase activity either by preventing the release of cytochrome c from the mitochondria and/or by binding to the apoptosis-activating factor (APAF-1). May attenuate inflammation by impairing NLRP1-inflammasome activation, hence CASP1 activation and IL1B release; BCL2 family
BCL2A1	ENSP000 00267953	Hemopoietic-specific early response protein; Retards apoptosis induced by IL-3 deprivation. May function in the response of hemopoietic cells to external signals and in maintaining endothelial survival during infection (By similarity). Can inhibit apoptosis induced by serum starvation in the mammary epithelial cell line HC11 (By similarity); BCL2 family
CDKN1A	ENSP000 00384849	Cyclin-dependent kinase inhibitor 1A (p21, Cip1); May be involved in p53/TP53 mediated inhibition of cellular proliferation in response to DNA damage. Binds to and inhibits cyclin-dependent kinase activity, preventing phosphorylation of critical cyclin-dependent kinase

		substrates and blocking cell cycle progression. Functions in the nuclear localization and assembly of cyclin D-CDK4 complex and promotes its kinase activity towards RB1. At higher stoichiometric ratios, inhibits the kinase activity of the cyclin D-CDK4 complex. Inhibits DNA synthesis by DNA polymerase delta by competing with POLD3 for PCNA binding.
CDKN2A	ENSP000 00418915	Cyclin-dependent kinase 4 inhibitor A; Acts as a negative regulator of the proliferation of normal cells by interacting strongly with CDK4 and CDK6. This inhibits their ability to interact with cyclins D and to phosphorylate the retinoblastoma protein.
CDKN2D	ENSP000 00377224	Cyclin-dependent kinase inhibitor 2D (p19, inhibits CDK4); Interacts strongly with CDK4 and CDK6 and inhibits them; Belongs to the CDKN2 cyclin-dependent kinase inhibitor family.
CR1	ENSP000 00356016	Complement component (3b/4b) receptor 1 (Knops blood group); Mediates cellular binding of particles and immune complexes that have activated complement; Blood group antigens
DLC1	ENSP000 00276297	StAR-related lipid transfer protein 12; Functions as a GTPase-activating protein for the small GTPases RHOA, RHOB, RHOC and CDC42, terminating their downstream signaling. This induces morphological changes and detachment through cytoskeletal reorganization, playing a critical role in biological processes such as cell migration and proliferation. Also functions in vivo as an activator of the phospholipase PLCD1. Active DLC1 increases cell migration velocity but reduces directionality.
DNMT1	ENSP000 00352516	DNA (cytosine-5-)-methyltransferase 1; Methylates CpG residues. Preferentially methylates hemimethylated DNA. Associates with DNA replication sites in S phase maintaining the methylation pattern in the newly synthesized strand, that is essential for epigenetic inheritance. Associates with chromatin during G2 and M phases to maintain DNA methylation independently of replication. It is responsible for maintaining methylation patterns established in development. DNA methylation is coordinated with methylation of histones. Mediates transcriptional repression by direct binding to HDAC2. In association with DNMT3B and via the recruitment of CTCFL/BORIS, involved in activation of BAG1 gene expression by modulating dimethylation of promoter histone H3 at H3K4 and H3K9. Probably forms a corepressor complex required for activated KRAS-mediated promoter hypermethylation and transcriptional silencing of tumor suppressor genes (TSGs) or other tumor-related genes in colorectal cancer (CRC) cells. Also required to maintain a transcriptionally repressive state of genes in undifferentiated embryonic stem cells (ESCs). Associates at promoter regions of tumor suppressor genes (TSGs) leading to their gene silencing. Promotes tumor growth; Seven-beta-strand methyltransferase motif containing
EGF	ENSP000 00265171	Pro-epidermal growth factor; EGF stimulates the growth of various epidermal and epithelial tissues in vivo and in vitro and of some fibroblasts in cell culture. Magnesiotropic hormone that stimulates magnesium reabsorption in the renal distal convoluted tubule via engagement of EGFR and activation of the magnesium channel TRPM6.

		Can induce neurite outgrowth in motoneurons of the pond snail Lymnaea stagnalis in vitro.
ETS1	ENSP000 00376436	V-ets avian erythroblastosis virus E26 oncogene homolog 1; Transcription factor. Directly controls the expression of cytokine and chemokine genes in a wide variety of different cellular contexts. May control the differentiation, survival and proliferation of lymphoid cells. May also regulate angiogenesis through regulation of expression of genes controlling endothelial cell migration and invasion.
FGF11	ENSP000 00293829	Fibroblast growth factor homologous factor 3; Probably involved in nervous system development and function.
FOS	ENSP000 00306245	FBJ murine osteosarcoma viral oncogene homolog; Nuclear phosphoprotein which forms a tight but non- covalently linked complex with the JUN/AP-1 transcription factor. In the heterodimer, FOS and JUN/AP-1 basic regions each seems to interact with symmetrical DNA half sites. On TGF-beta activation, forms a multimeric SMAD3/SMAD4/JUN/FOS complex at the AP1/SMAD- binding site to regulate TGF-beta-mediated signaling. Has a critical function in regulating the development of cells destined to form and maintain the skeleton. It is thought to have an important role in signal transduction, cell proliferation and differentiation. In growing cells, activates phospholipid synthesis, possibly by activating CDS1 and PI4K2A. This activity requires Tyr-dephosphorylation and association with the endoplasmic reticulum; Basic leucine zipper proteins
GFI1	ENSP000 00359357	Growth factor independent 1 transcription repressor; Transcription repressor essential for hematopoiesis. Functions in a cell-context and development-specific manner. Binds to 5'-TAAATCAC[AT]GCA-3' in the promoter region of a large number of genes. Component of several complexes, including the EHMT2- GFI1-HDAC1, AJUBA-GFI1-HDAC1 and RCOR-GFI-KDM1A-HDAC complexes, that suppress, via histone deacetylase (HDAC) recruitment, a number of genes implicated in multilineage blood cell development. Regulates neutrophil differentiation, promotes proliferation of lymphoid cells, and is required for granulocyte development. Mediates, together with U2AF1L4, the alternative splicing of CD45 and controls T-cell receptor signaling. Regulates the endotoxin- mediated Toll-like receptor (TLR) inflammatory response by antagonizing RELA. Cooperates with CBFA2T2 to regulate ITGB1- dependent neurite growth. Controls cell-cycle progression by repressing CDKNIA/p21 transcription in response to TGFB1 via recruitment of GFI1 by ZBTB17 to the CDKNIA/p21 and CDKNIB promoters. Required for the maintenance of inner ear hair cells; SNAG transcriptional repressors
GNA11	ENSP000 00078429	Guanine nucleotide binding protein (G protein), alpha 11 (Gq class); Guanine nucleotide-binding proteins (G proteins) are involved as modulators or transducers in various transmembrane signaling systems. Acts as an activator of phospholipase C; G protein subunits alpha, group q
HSPA2	ENSP000 00378199	Heat shock-related 70 kDa protein 2; Molecular chaperone implicated in a wide variety of cellular processes, including protection of the proteome from stress, folding and transport of newly synthesized polypeptides, activation of proteolysis of misfolded proteins and the

		formation and dissociation of protein complexes. Plays a pivotal role in the protein quality control system, ensuring the correct folding of proteins, the re-folding of misfolded proteins and controlling the targeting of proteins for subsequent degradation. This is achieved through cycles of ATP binding, ATP hydrolysis and ADP release, mediated by co-chaperones. The affinity for polypeptides is regulated by its nucleotide bound state. In the ATP-bound form, it has a low affinity for substrate proteins. However, upon hydrolysis of the ATP to ADP, it undergoes a conformational change that increases its affinity for substrate proteins. It goes through repeated cycles of ATP hydrolysis and nucleotide exchange, which permits cycles of substrate binding and release. Plays a role in spermatogenesis. In association with SHCBP1L may participate in the maintenance of spindle integrity during meiosis in male germ cells (By similarity); Heat shock 70kDa proteins
ID1	ENSP00000365280	Inhibitor of DNA binding 1, dominant negative helix-loop-helix protein; Transcriptional regulator (lacking a basic DNA binding domain) which negatively regulates the basic helix-loop-helix (bHLH) transcription factors by forming heterodimers and inhibiting their DNA binding and transcriptional activity. Implicated in regulating a variety of cellular processes, including cellular growth, senescence, differentiation, apoptosis, angiogenesis, and neoplastic transformation. Inhibits skeletal muscle and cardiac myocyte differentiation. Regulates the circadian clock by repressing the transcriptional activator activity of the CLOCK-ARNTL/BMAL1 heterodimer (By similarity).
ID2	ENSP00000234091	Inhibitor of DNA binding 2, dominant negative helix-loop-helix protein; Transcriptional regulator (lacking a basic DNA binding domain) which negatively regulates the basic helix-loop-helix (bHLH) transcription factors by forming heterodimers and inhibiting their DNA binding and transcriptional activity. Implicated in regulating a variety of cellular processes, including cellular growth, senescence, differentiation, apoptosis, angiogenesis, and neoplastic transformation. Inhibits skeletal muscle and cardiac myocyte differentiation. Regulates the circadian clock by repressing the transcriptional activator activity of the CLOCK-ARNTL/BMAL1 heterodimer. Restricts the CLOCK and ARNTL/BMAL1 localization to the cytoplasm. Plays a role in both the input and output pathways of the circadian clock: in the input component, is involved in modulating the magnitude of photic entrainment and in the output component, contributes to the regulation of a variety of liver clock-controlled genes involved in lipid metabolism.
IL12B	ENSP00000231228	Cytotoxic lymphocyte maturation factor 40 kDa subunit; Cytokine that can act as a growth factor for activated T and NK cells, enhance the lytic activity of NK/lymphokine- activated killer cells, and stimulate the production of IFN-gamma by resting PBMC; Belongs to the type I cytokine receptor family. Type 3 subfamily.
ITGA9	ENSP00000264741	Integrin alpha-RLC; Integrin alpha-9/beta-1 (ITGA9:ITGB1) is a receptor for VCAM1, cytotactin and osteopontin. It recognizes the sequence A-E-I-D-G-I-E-L in cytotactin.
KL	ENSP00000369442	Klotho; May have weak glycosidase activity towards glucuronylated steroids. However, it lacks essential active site Glu residues at positions 239 and 872, suggesting it may be inactive as a glycosidase in vivo. May

		be involved in the regulation of calcium and phosphorus homeostasis by inhibiting the synthesis of active vitamin D (By similarity). Essential factor for the specific interaction between FGF23 and FGFR1 (By similarity); Belongs to the glycosyl hydrolase 1 family. Klotho subfamily.
KMT2E	ENSP00000312379	Myeloid/lymphoid or mixed-lineage leukemia protein 5; Histone methyltransferase that specifically mono- and dimethylates 'Lys-4' of histone H3 (H3K4me1 and H3K4me2). H3 'Lys- 4' methylation represents a specific tag for epigenetic transcriptional activation. Key regulator of hematopoiesis involved in terminal myeloid differentiation and in the regulation of hematopoietic stem cell (HSCs) self-renewal by a mechanism that involves DNA methylation. Plays an essential role in retinoic- acid-induced granulopoiesis by acting as a coactivator of RAR-alpha (RARA) in target gene promoters. Also acts as an important cell cycle regulator, participating in cell cycle regulatory network machinery at multiple cell cycle stages. Required to suppress inappropriate expression of S-phase-promoting genes and maintain expression of determination genes in quiescent cells. Overexpression inhibits cell cycle progression, while knockdown induces cell cycle arrest at both the G1 and G2/M phases; Belongs to the class V-like SAM-binding methyltransferase superfamily. Histone-lysine methyltransferase family. TRX/MLL subfamily.
LIG4	ENSP00000484288	Polydeoxyribonucleotide synthase [ATP] 4; Efficiently joins single-strand breaks in a double- stranded polydeoxynucleotide in an ATP-dependent reaction. Involved in DNA non-homologous end joining (NHEJ) required for double-strand break repair and V(D)J recombination. The LIG4-XRCC4 complex is responsible for the NHEJ ligation step, and XRCC4 enhances the joining activity of LIG4. Binding of the LIG4-XRCC4 complex to DNA ends is dependent on the assembly of the DNA- dependent protein kinase complex DNA-PK to these DNA ends.
LMX1B	ENSP00000347684	LIM homeobox transcription factor 1, beta; Essential for the specification of dorsal limb fate at both the zeugopodal and autopodal levels; LIM class homeoboxes
MAPK10	ENSP00000352157	Stress-activated protein kinase JNK3; Serine/threonine-protein kinase involved in various processes such as neuronal proliferation, differentiation, migration and programmed cell death. Extracellular stimuli such as proinflammatory cytokines or physical stress stimulate the stress- activated protein kinase/c-Jun N-terminal kinase (SAP/JNK) signaling pathway. In this cascade, two dual specificity kinases MAP2K4/MKK4 and MAP2K7/MKK7 phosphorylate and activate MAPK10/JNK3. In turn, MAPK10/JNK3 phosphorylates a number of transcription factors, primarily components of AP-1 such as JUN and ATF2 and thus regulates AP-1 transcriptional activity. Plays regulatory roles in the signaling pathways during neuronal apoptosis. Phosphorylates the neuronal microtubule regulator STMN2. Acts in the regulation of the amyloid-beta precursor protein/APP signaling during neuronal differentiation by phosphorylating APP. Participates also in neurite growth in spiral ganglion neurons. Phosphorylates the CLOCK-

		ARNTL/BMAL1 heterodimer and plays a role in the photic regulation of the circadian clock.
MET	ENSP00000317272	MET proto-oncogene, receptor tyrosine kinase; Receptor tyrosine kinase that transduces signals from the extracellular matrix into the cytoplasm by binding to hepatocyte growth factor/HGF ligand. Regulates many physiological processes including proliferation, scattering, morphogenesis and survival. Ligand binding at the cell surface induces autophosphorylation of MET on its intracellular domain that provides docking sites for downstream signaling molecules. Following activation by ligand, interacts with the PI3-kinase subunit PIK3R1, PLCG1, SRC, GRB2, STAT3 or the adapter GAB1. Recruitment of these downstream effectors by MET leads to the activation of several signaling cascades including the RAS-ERK, PI3 kinase-AKT, or PLCgamma-PKC. The RAS-ERK activation is associated with the morphogenetic effects while PI3K/AKT coordinates prosurvival effects. During embryonic development, MET signaling plays a role in gastrulation, development and migration of muscles and neuronal precursors, angiogenesis and kidney formation. In adults, participates in wound healing as well as organ regeneration and tissue remodeling. Promotes also differentiation and proliferation of hematopoietic cells. May regulate cortical bone osteogenesis (By similarity).
MLF1	ENSP00000376568	Myelodysplasia-myeloid leukemia factor 1; Involved in lineage commitment of primary hemopoietic progenitors by restricting erythroid formation and enhancing myeloid formation. Interferes with erythropoietin-induced erythroid terminal differentiation by preventing cells from exiting the cell cycle through suppression of CDKN1B/p27Kip1 levels. Suppresses RFWD2/COP1 activity via CSN3 which activates p53 and induces cell cycle arrest. Binds DNA and affects the expression of a number of genes so may function as a transcription factor in the nucleus; Belongs to the MLF family.
NFKB1	ENSP00000226574	Nuclear factor of kappa light polypeptide gene enhancer in B-cells 1; NF-kappa-B is a pleiotropic transcription factor present in almost all cell types and is the endpoint of a series of signal transduction events that are initiated by a vast array of stimuli related to many biological processes such as inflammation, immunity, differentiation, cell growth, tumorigenesis and apoptosis. NF-kappa-B is a homo- or heterodimeric complex formed by the Rel-like domain-containing proteins RELA/p65, RELB, NFKB1/p105, NFKB1/p50, REL and NFKB2/p52 and the heterodimeric p65-p50 complex appears to be most abundant one. The dimers bind at kappa-B sites in the DNA of their target genes and the individual dimers have distinct preferences for different kappa-B sites that they can bind with distinguishable affinity and specificity. Different dimer combinations act as transcriptional activators or repressors, respectively. NF-kappa-B is controlled by various mechanisms of post-translational modification and subcellular compartmentalization as well as by interactions with other cofactors or corepressors. NF-kappa-B complexes are held in the cytoplasm in an inactive state complexed with members of the NF-kappa-B inhibitor (I-kappa-B) family. In a conventional activation pathway, I-kappa-B is phosphorylated by I-kappa-B kinases (IKKs) in response to different activators, subsequently

		degraded thus liberating the active NF-kappa-B complex which translocates to the nucleus. NF-kappa-B heterodimeric p65-p50 and RelB-p50 complexes are transcriptional activators. The NF-kappa-B p50-p50 homodimer is a transcriptional repressor, but can act as a transcriptional activator when associated with BCL3. NFKB1 appears to have dual functions such as cytoplasmic retention of attached NF-kappa-B proteins by p105 and generation of p50 by a cotranslational processing. The proteasome-mediated process ensures the production of both p50 and p105 and preserves their independent function, although processing of NFKB1/p105 also appears to occur post-translationally. p50 binds to the kappa-B consensus sequence 5'-GGRNNYYCC-3', located in the enhancer region of genes involved in immune response and acute phase reactions. In a complex with MAP3K8, NFKB1/p105 represses MAP3K8-induced MAPK signaling; active MAP3K8 is released by proteasome-dependent degradation of NFKB1/p105.
NOTCH1	ENSP00000277541	Translocation-associated notch protein TAN-1; Functions as a receptor for membrane-bound ligands Jagged1, Jagged2 and Delta1 to regulate cell-fate determination. Upon ligand activation through the released notch intracellular domain (NICD) it forms a transcriptional activator complex with RBPJ/RBPSUH and activates genes of the enhancer of split locus. Affects the implementation of differentiation, proliferation and apoptotic programs. Involved in angiogenesis; negatively regulates endothelial cell proliferation and migration and angiogenic sprouting. Involved in the maturation of both CD4+ and CD8+ cells in the thymus. Important for follicular differentiation and possibly cell fate selection within the follicle. During cerebellar development, functions as a receptor for neuronal DNER and is involved in the differentiation of Bergmann glia. Represses neuronal and myogenic differentiation. May play an essential role in postimplantation development, probably in some aspect of cell specification and/or differentiation. May be involved in mesoderm development, somite formation and neurogenesis. May enhance HIF1A function by sequestering HIF1AN away from HIF1A. Required for the THBS4 function in regulating protective astrogenesis from the subventricular zone (SVZ) niche after injury. Involved in determination of left/right symmetry by modulating the balance between motile and immotile (sensory) cilia at the left-right organiser (LRO); Ankyrin repeat domain containing
PRKAA2	ENSP00000360290	5'-AMP-activated protein kinase catalytic subunit alpha-2; Catalytic subunit of AMP-activated protein kinase (AMPK), an energy sensor protein kinase that plays a key role in regulating cellular energy metabolism. In response to reduction of intracellular ATP levels, AMPK activates energy-producing pathways and inhibits energy-consuming processes: inhibits protein, carbohydrate and lipid biosynthesis, as well as cell growth and proliferation. AMPK acts via direct phosphorylation of metabolic enzymes, and by longer-term effects via phosphorylation of transcription regulators. Also acts as a regulator of cellular polarity by remodeling the actin cytoskeleton; probably by indirectly activating myosin. Regulates lipid synthesis by phosphorylating and inactivating lipid metabolic enzymes such as ACACA, ACACB, GYS1, HMGCR and

		LIPE; regulates fatty acid and cholesterol synthesis by phosphorylating acetyl-CoA carboxylase (ACACA and ACACB) and hormone-sensitive lipase (LIPE) enzymes, respectively. Regulates insulin-signaling and glycolysis by phosphorylating IRS1, PFKFB2 and PFKFB3. Involved in insulin receptor/INSR internalization. AMPK stimulates glucose uptake in muscle by increasing the translocation of the glucose transporter SLC2A4/GLUT4 to the plasma membrane, possibly by mediating phosphorylation of TBC1D4/AS160. Regulates transcription and chromatin structure by phosphorylating transcription regulators involved in energy metabolism such as CRTC2/TORC2, FOXO3, histone H2B, HDAC5, MEF2C, MLXIPL/ChREBP, EP300, HNF4A, p53/TP53, SREBF1, SREBF2 and PPARGC1A. Acts as a key regulator of glucose homeostasis in liver by phosphorylating CRTC2/TORC2, leading to CRTC2/TORC2 sequestration in the cytoplasm. In response to stress, phosphorylates 'Ser-36' of histone H2B (H2BS36ph), leading to promote transcription. Acts as a key regulator of cell growth and proliferation by phosphorylating TSC2, RPTOR and ATG1/ULK1: in response to nutrient limitation, negatively regulates the mTORC1 complex by phosphorylating RPTOR component of the mTORC1 complex and by phosphorylating and activating TSC2. In response to nutrient limitation, promotes autophagy by phosphorylating and activating ATG1/ULK1. AMPK also acts as a regulator of circadian rhythm by mediating phosphorylation of CRY1, leading to destabilize it. May regulate the Wnt signaling pathway by phosphorylating CTNNB1, leading to stabilize it. Also phosphorylates CFTR, EEF2K, KLC1, NOS3 and SLC12A1. Plays an important role in the differential regulation of pro-autophagy (composed of PIK3C3, BECN1, PIK3R4 and UVRAG or ATG14) and non-autophagy (composed of PIK3C3, BECN1 and PIK3R4) complexes, in response to glucose starvation. Can inhibit the non-autophagy complex by phosphorylating PIK3C3 and can activate the pro-autophagy complex by phosphorylating BECN1 (By similarity); Belongs to the protein kinase superfamily. CAMK Ser/Thr protein kinase family. SNF1 subfamily.
RELN	ENSP00000392423	Reelin; Extracellular matrix serine protease that plays a role in layering of neurons in the cerebral cortex and cerebellum. Regulates microtubule function in neurons and neuronal migration. Affects migration of sympathetic preganglionic neurons in the spinal cord, where it seems to act as a barrier to neuronal migration. Enzymatic activity is important for the modulation of cell adhesion. Binding to the extracellular domains of lipoprotein receptors VLDLR and LRP8/APOER2 induces tyrosine phosphorylation of DAB1 and modulation of TAU phosphorylation (By similarity); Belongs to the reelin family.
RUNX1T1	ENSP00000402257	Runt-related transcription factor 1; translocated to, 1 (cyclin D-related); Transcriptional corepressor which facilitates transcriptional repression via its association with DNA-binding transcription factors and recruitment of other corepressors and histone-modifying enzymes. Can repress the expression of MMP7 in a ZBTB33-dependent manner. Can repress transactivation mediated by TCF12. Acts as a negative regulator of adipogenesis (By similarity). The AML1-MTG8/ETO fusion protein frequently found in leukemic cells is involved in leukemogenesis and

		contributes to hematopoietic stem/progenitor cell self-renewal; Zinc fingers MYND-type
SHH	ENSP00000297261	Shh unprocessed N-terminal signaling and C-terminal autoprocessing domains; Sonic hedgehog protein: The C-terminal part of the sonic hedgehog protein precursor displays an autoproteolysis and a cholesterol transferase activity (By similarity). Both activities result in the cleavage of the full-length protein into two parts (ShhN and ShhC) followed by the covalent attachment of a cholesterol moiety to the C-terminal of the newly generated ShhN (By similarity). Both activities occur in the reticulum endoplasmic (By similarity). Once cleaved, ShhC is degraded in the endoplasmic reticulum (By similarity); Hedgehog signaling molecule family
SPP1	ENSP00000378517	Secreted phosphoprotein 1; Binds tightly to hydroxyapatite. Appears to form an integral part of the mineralized matrix. Probably important to cell-matrix interaction; Endogenous ligands
STAT3	ENSP00000264657	Signal transducer and activator of transcription 3 (acute-phase response factor); Signal transducer and transcription activator that mediates cellular responses to interleukins, KITLG/SCF, LEP and other growth factors. Once activated, recruits coactivators, such as NCOA1 or MED1, to the promoter region of the target gene. May mediate cellular responses to activated FGFR1, FGFR2, FGFR3 and FGFR4. Binds to the interleukin-6 (IL-6)-responsive elements identified in the promoters of various acute-phase protein genes. Activated by IL31 through IL31RA. Acts as a regulator of inflammatory response by regulating differentiation of naive CD4(+) T-cells into T-helper Th17 or regulatory T-cells (Treg): deacetylation and oxidation of lysine residues by LOXL3, leads to disrupt STAT3 dimerization and inhibit its transcription activity. Involved in cell cycle regulation by inducing the expression of key genes for the progression from G1 to S phase, such as CCND1. Mediates the effects of LEP on melanocortin production, body energy homeostasis and lactation (By similarity). May play an apoptotic role by transctivating BIRC5 expression under LEP activation. Cytoplasmic STAT3 represses macroautophagy by inhibiting EIF2AK2/PKR activity. Plays a crucial role in basal beta cell functions, such as regulation of insulin secretion (By similarity); SH2 domain containing
TNFRSF10B	ENSP00000276431	Tumor necrosis factor receptor superfamily, member 10b; Receptor for the cytotoxic ligand TNFSF10/TRAIL. The adapter molecule FADD recruits caspase-8 to the activated receptor. The resulting death-inducing signaling complex (DISC) performs caspase-8 proteolytic activation which initiates the subsequent cascade of caspases (aspartate-specific cysteine proteases) mediating apoptosis. Promotes the activation of NF-kappa-B. Essential for ER stress-induced apoptosis; CD molecules
TUBB3	ENSP00000320295	Tubulin, beta 3 class III; Tubulin is the major constituent of microtubules. It binds two moles of GTP, one at an exchangeable site on the beta chain and one at a non-exchangeable site on the alpha chain. TUBB3 plays a critical role in proper axon guidance and mantainance; Belongs to the tubulin family.
