# Supplementary material for: Gene Expression Profiles Identify Biomarkers of Resistance to Decitabine in Myelodysplastic Syndromes
Source: Cells. 2021 Dec 10;10(12):3494. doi: 10.3390/cells10123494 (PMC8700444; doi:10.3390/cells10123494)
Supplement: Supplementary file 1 [file cells-10-03494-s001.zip › Table S6_38 genes in bone marrow.pdf]

Table S6. Expression levels of 38 genes in bone marrow from MDS patients.

Gene Name	Patients ($2^{-\Delta\Delta CT}$)			
	1	2	3	4
TUBB3	2.11	1.14	0.65	1.53
SHH	0.62	0.64	0.45	0.07
AMER1	0.44	0.94	0.61	0.86
LMX1B	0.80	1.37	0.63	0.56
GFI1	1.27	1.84	0.92	0.77
IL12B	1.58	1.32	0.85	0.75
ETS1	1.66	0.70	0.66	0.83
HSPA2	1.07	0.56	0.99	0.81
RELN	0.17	1.49	0.42	0.62
AKT3	15.94	1.95	4.98	98.37
STAT3	1.62	1.08	0.82	4.24
ID2	1.05	0.96	0.87	1.20
CDKN2A	1.00	0.52	0.66	0.68
GNA11	0.68	0.83	0.87	0.58
BCL2	1.28	0.90	1.35	0.61
ID1	1.17	1.33	0.52	0.55
MAPK10	1.28	0.97	0.92	0.60
KMT2E	0.40	1.13	0.40	0.64
DLC1	1.13	1.03	0.73	0.72
PRKAA2	1.08	1.26	1.28	0.80
SPP1	0.67	1.03	0.83	0.43
CDKN2D	1.12	0.58	0.97	5.25
DNMT1	0.52	1.15	0.53	0.8
NOTCH1	2.76	0.70	1.09	1.63
ITGA9	0.39	1.86	0.32	0.79
FGF11	0.18	1.03	0.20	0.19
KL	0.73	1.04	0.85	0.57
MET	1.52	1.67	1.14	0.86
CR1	2.44	0.99	0.75	1.53
CDKN1A	1.10	0.91	0.95	0.95
TNFRSF10B	0.13	1.28	0.12	0.17
NFKB1	2.12	1.98	1.41	1.34
FOS	10.82	0.89	4.51	9.05
MLF1	1.33	1.48	0.88	0.79
BCL2A1	1.04	1.09	1.02	0.80
RUNX1T1	0.98	1.15	0.93	0.90
EGF	1.79	1.58	3.20	2.61
LIG4	1.10	0.98	0.85	0.62

All expression were performed in triplicate, and value are significant at the $p < 0.05$ level.
